# *Pouteria lucuma* Pulp and Skin: In Depth Chemical Profile and Evaluation of Antioxidant Activity

**DOI:** 10.3390/molecules26175236

**Published:** 2021-08-29

**Authors:** Milena Masullo, Antonietta Cerulli, Cosimo Pizza, Sonia Piacente

**Affiliations:** Dipartimento di Farmacia, Università degli Studi di Salerno, via Giovanni Paolo II n. 132, 84084 Fisciano, SA, Italy; mmasullo@unisa.it (M.M.); acerulli@unisa.it (A.C.); pizza@unisa.it (C.P.)

**Keywords:** *Pouteria lucuma*, LC-ESI/LTQOrbitrap/MS, flavonoids, polar lipids, antioxidant activity

## Abstract

*Pouteria lucuma* Ruiz and Pav., known as the ‘Gold of the Incas’ or ‘lucuma’, is a subtropical fruit belonging to the Sapotaceae family, with a very sweet flavor, used to prepare cakes, ice creams as well as in the baking and dairy industries. To date, the content of primary metabolites is known, but little information is reported about the composition in specialized metabolites. Moreover, no study is reported on skin which represent an important agricultural waste due to the high demand for lucuma. In order to have a preliminary metabolite profile of *Pouteria lucuma*, the extracts of pulp and skin have been analyzed by LC-ESI/LTQOrbitrap/MS/MS in negative ion mode. The careful analysis of the accurate masses, of the molecular formulas and of the ESI/MS spectra allowed to identify specialized metabolites belonging to phenolic, flavonoid and polar lipid classes. The LC-MS/MS analysis guided the isolation of compounds occurring in the pulp extract whose structures have been characterized by spectroscopic methods including 1D- and 2D-NMR experiments and ESI-MS analysis. Furthermore, the phenolic content of the extracts along with the antioxidant activity of extracts and isolated compounds was evaluated.

## 1. Introduction

The genus *Pouteria* is a pan tropical group consisting of 325 species, many of which produce high-quality timber and edible fruit, represent great economic value. In addition to their commercial significance, several species have been used in folk medicine for several purposes, due to their biological activities including antioxidant, anti-inflammatory, antibacterial and antifungal properties [1,2]. 

*Pouteria lucuma* Ruiz and Pav. (Sapotaceae) is a subtropical fruit known as the ‘Gold of the Incas’ or ‘lucuma’ which is native to the Andean region and is found in Peru, Chile and Ecuador [3,4]. *P. lucuma* was a food of ancient cultivation [5], as suggested by ceramic representations of lucuma dating back to the Nazca and Moche pre-Inca civilizations. Lucuma has an ovoid or elliptical shape depending on the cultivar, a diameter variable between 7.5 and 10 cm, thin green or yellow-green skin and sweet yellow-orange flesh. Lucuma possesses a sweet taste and wonderful flavor and aroma described as ‘caramel like, maple-like with a bit of pumpkin-like taste’ [5]. For these characteristics, it is used to prepare cakes, ice creams and is mainly processed into frozen fruit or pulp and flour and then used in the baking and dairy industries both in Peru and in other countries such as the USA and members of the European Union. In Peru and Chile, ‘lucuma’ flavor ice cream is very popular. Recently, lucuma has attracted the attention of researchers because it constitutes a source of various compounds of interest for their antioxidant properties such as carotenoids and phenolic compounds [3,5,6]. Therefore, the taste along with its beneficial properties gained the attention of consumers and increased the trade of lucuma, which is widely distributed also in online markets. There are few studies focused on the content of this fruit [3,5,6,7], and no studies have been reported on its skin, which represents an important agricultural waste due to the high demand for lucuma. 

Herein, the total phenolic content of pulp and skin extracts was determined by Folin-Ciocalteu assay and the antioxidant activity was evaluated by 2,2-diphenyl-1-picrylhydrazyl (DPPH^•^) and trolox equivalent antioxidant capacity (TEAC) assays. The *n*-BuOH extract of the pulp and the methanol extract of the skin of *P. lucuma* fruits were preliminarily investigated by liquid chromatography coupled to high resolution mass spectrometry (ESI-Orbitrap-MS) in negative ion mode. The high resolution mass spectrometry is an advanced and informative analytical technique used for the metabolite profiling of plant extracts [8,9], and so far HR-LC-MS has not been employed for the chemical investigation of lucuma pulp. A recent investigation of lucuma seeds, aimed at valorizing agricultural industry waste, was performed by LC-MS/MS. Compounds tentatively identified as aminoacids, organic acids, nucleosides, phenolic acids, phenolic alcohols, flavonoids, and lipids along with some unknown compounds were reported [10]. Previous investigations on lucuma mainly reported the identification of primary metabolites [3,5,6] and the analysis of different biotypes, highlighting differences in the content of sugars, organic acids, total phenolics, total carotenoids while few phenolic compounds were identified by HPLC-DAD [3,5]. Moreover, volatile compounds responsible for the lucuma aroma were identified [6,11]. To the best of our knowledge, the chemical composition of the skin of lucuma has never been investigated before. Therefore, this work presents the first comprehensive metabolite profiling of *P. lucuma* pulp and skin polar extracts performed by an advanced technique like HR-LC-ESI-Orbitrap-MS along with NMR spectroscopy that provides an unambiguous structural elucidation of compounds. The LC-MS profile guided the isolation of compounds occurring in the pulp extract, of which the structures were elucidated by 1D- and 2D-NMR experiments. Finally, the antioxidant activity of isolated phenolic compounds was also investigated. 

## 2. Results and Discussion

### 2.1. Evaluation of the Total Phenolic Content and Antioxidant Activity of P. lucuma Pulp and Skin

To evaluate the total phenolic content in lucuma extracts, a Folin-Ciocalteu assay was carried out [12]. For the *n*-BuOH extract of the pulp and the methanol extract of the skin, the total phenolic content was determined using the regression equation y = 0.0027x + 0.0982 (R^2^ = 0.993) where x is the concentration of gallic acid (μg/mL) and y is the absorbance measured at 760 nm; the results are reported in Table 1. *n*-BuOH extract of the pulp showed six times lower phenolic content in comparison to the methanol extract of the skin. The antioxidant activity of the extracts was evaluated by DPPH assay. For tested extracts, the inhibitory concentration (IC_50_) value was calculated and the results are reported in Table 1. The methanol extract of skin exerted a stronger antioxidant activity if compared to the *n*-BuOH extract of the pulp. Moreover, ABTS^•+^ radical scavenging activity was carried out [13]. Standard Trolox solutions were employed to obtain the calibration curve, resulted to be y = 20.345x + 1.2234 (R^2^ = 0.998). The extracts were then tested, and the obtained results were used to evaluate their antioxidant activity, expressed as TEAC, defined as the concentration (mM) of a standard Trolox solution exerting the same antioxidant activity of a 1 mg/mL solution of the tested extract. 

Additionally, in this case the antioxidant activity evaluated by this method showed a higher activity for the skin extract than the pulp extract.

Comparing the obtained results with those reported in the literature, the total phenolic content of the pulp as well as the antioxidant activity are in agreement with those reported for some lucuma varieties [5]. Regarding the skin, this is the first report of the total phenolic content and the antioxidant activity. In the literature, a recent work reported their investigation of the seeds aimed at valorizing this waste [10]. Of note also is that the lucuma skin could represent an interesting by-product which is useful for the preparation of food supplements, considering that skin showed a total phenolic content as well as a DPPH and TEAC activity higher than seeds [10].

### 2.2. LC-MS Analysis of Specialized Metabolites Occurring in P. lucuma Pulp 

**Figure 1:**
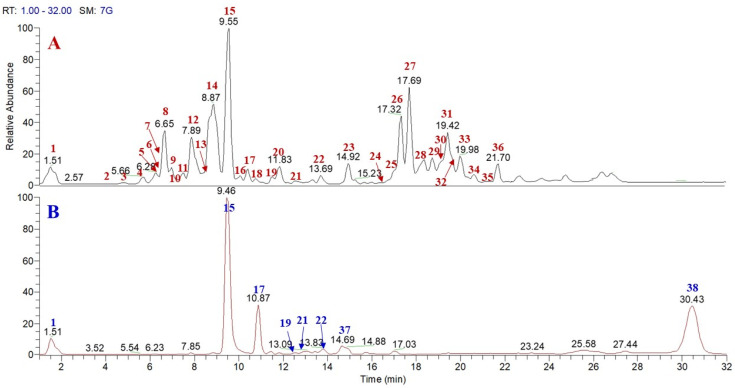
HR-LC-ESI-Orbitrap-MS profile of pulp *n*-BuOH extract (**A**) and skin methanol extract (**B**) of *P. lucuma*.

Preliminary metabolite profiles of pulp *n*-BuOH extract of *P. lucuma* and skin MeOH extract were obtained by HR-LC-ESI-Orbitrap-MS analysis in negative ion mode. The LC-MS profile of *n*-BuOH extract of pulp (Figure 1) showed 36 main peaks corresponding to flavonoids, phenolics, as well as polar lipids derivatives. HR-LC-ESI-Orbitrap-MS/MS experiments using “data dependent scan” mode were performed by acquiring MS/MS spectra of the first and the second most intense ions produced during the HRMS. Some of the main peaks were tentatively attributed according to the accurate masses, characteristic fragmentation patterns, and retention times and by comparison with the literature data on *P. lucuma*. In detail, compounds **1**, **6**, **8**, and **14** showed a fragmentation pattern typically ascribable to phenolic derivatives in which the main product ion (at *m*/*z* 169, 163, 193, and 227, respectively) was generated by the neutral loss of 162 Da ascribable to a hexose unit. Therefore, compounds **1**, **6**, **8**, and **14** were tentatively identified as galloyl glycoside, *p*-coumaroyl glycoside, *p*-feruloyl glycoside, and resveratrol glycoside, respectively.

Furthermore, LC-MS analysis highlighted some flavonoids tentatively identified as belonging to the class of flavan-3-ols: gallocatechin (**2**), epigallocatechin (**5**), catechin (**7**), epicatechin (**10**), gallocatechin gallate (**11**); dihydroflavonols: ampelopsin (**12**) and taxifolin (**15**), as well as flavonols (**13**, **16** and **19**) [14]. Glycosylated flavonols were identified according to the fragmentation patterns evidencing the loss of 146 Da, corresponding to a deoxyhexose unit; in this way, compounds **13** and **16** were determined as myricetin-*O*-deoxyglycoside and quercetin-*O*-deoxyglycoside, respectively.

**Figure 2:**
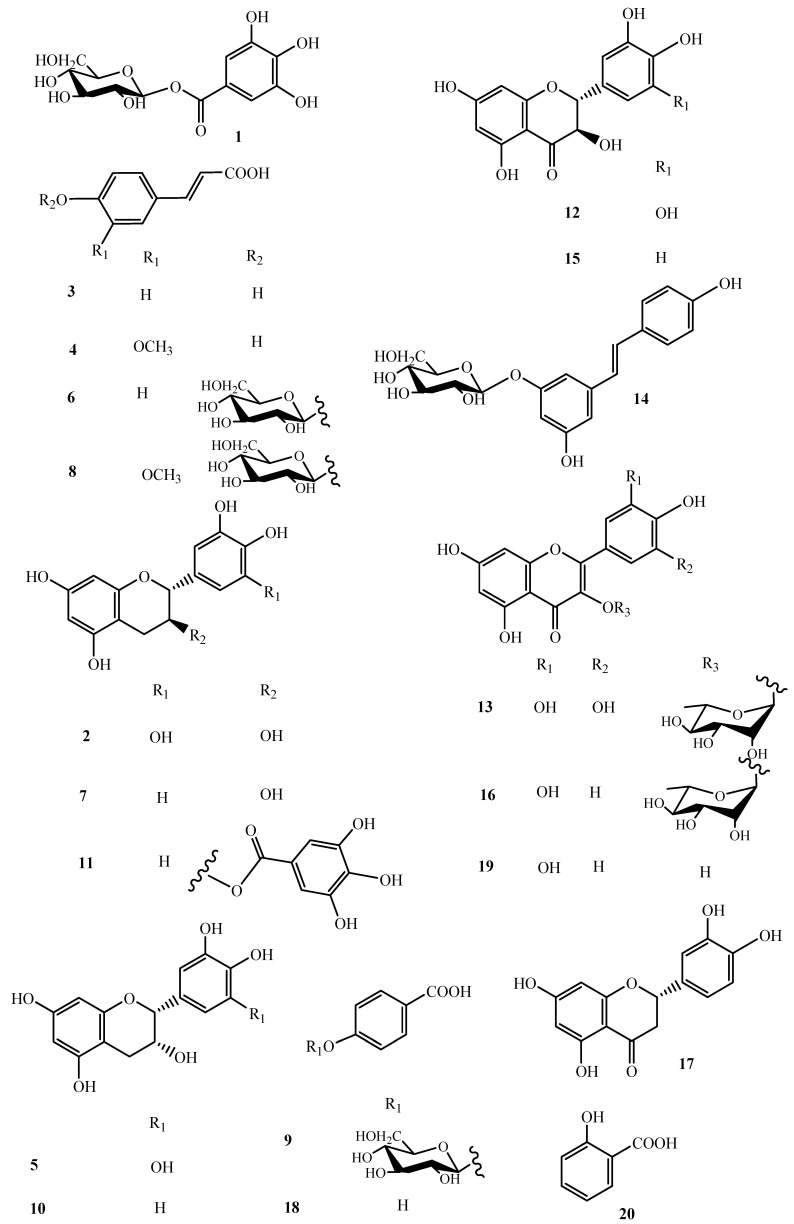
Compounds isolated from *P. lucuma* pulp *n*-BuOH extract.

With the aim to obtain an in depth knowledge of the polar constituents of the pulp and to unambiguously attribute the peaks occurring in the HR-LC-ESI-Orbitrap-MS profile, a phytochemical investigation of the pulp *n*-BuOH extract was performed. *n*-BuOH extract was fractionated directly by HPLC-UV (Figure S1) to afford pure compounds, of which the structures were elucidated by 1D- and 2D-NMR experiments (Figure 2 and Figures S2–S21). In this way, galloyl 1-*O*-glucopyranoside (**1**), gallocatechin (**2**), *p*-coumaric acid (**3**), *p*-ferulic acid (**4**), epigallocatechin (**5**), *p*-coumaroyl 4-*O*-β-d-glucopyranoside (**6**), catechin (**7**), *p*-feruloyl-4-*O*-β-d-glucopyranoside (**8**), 4-hydroxybenzoic acid 4-*O*-β-d-glucopyranoside (**9**), epicatechin (**10**), gallocatechin-gallate (**11**), ampelopsin (**12**), myricetin 3-*O*-α-l-rhamnopyranoside (**13**), resveratrol-3-*O*-β-d-glucopyranoside (**14**), taxifolin (**15**), quercetin-3-*O*-α-l-rhamnopyranoside (**16**), eriodictyol (**17**), *p*-hydroxy benzoic acid (**18**), quercetin (**19**) and salycilic acid (**20**) were identified by comparison of their spectroscopic data with those reported in the literature [15,16,17,18,19,20].

In previous reports, some derivatives of gallocatechin, epigallocatechin, catechin and epicatechin, gallic acid, ellagic acid and derivative of hesperetin were only tentatively identified in lucuma on the basis of their UV spectra [3,5], thus this is the first report of their exact structures by isolation and structural elucidation. Eriodictyol (**17**) and quercetin (**19**) were reported in lucuma seeds [10]. Many compounds (**1**, **3**, **4**, **6**, **8**, **9**, **11**–**19**), including salycilic acid (**20**), are reported here in lucuma pulp for the first time.

### 2.3. LC-MS Qualitative Analysis of Polar Lipids in P. lucuma Pulp n-BuOH Extract

#### 2.3.1. Identification of Oxylipins (**21**–**25**)

The analysis of the LC-ESI/LTQOrbitrap/MS spectra in negative ionization mode of the *n*-BuOH pulp extract showed pseudomolecular ions characterized by a molecular formula C_18_H_36−2*n*_O_2*m*_ with *n* = 1, 2, 3 or 4 and *m* = 1, 2 or 3 and a fragmentation pattern attributable to the class of oxylipins (Table 2 and Table S1). These compounds are hydroxy fatty acids deriving from the oxidative metabolism of PUFAs such as linolenic acid (ALA, C18:3) and linoleic acid (LA, C18:2), differing in the unsaturation degree and number of hydroxyl groups [21,22]. Oxylipins yielded highly diagnostic MS^2^ fragmentation patterns (Table 2). According to the literature data [19,23], the position on the acyl chain of both hydroxyl groups and double bonds could be putatively assigned on the basis of characteristic product ions and diagnostic neutral losses generated by molecular rearrangements involving the head and the end of the acyl chain, respectively. Product ions generated by one or more consecutive neutral losses of 18 Da allowed to ascertain the number of hydroxyl groups occurring in the oxylipin structure. The detection of characteristic product ions, such as those at nominal *m*/*z* 171 (C_9_H_15_O_3_) and *m*/*z* 201 (C_10_H_17_O_4_), or those at nominal *m*/*z* 229 (C_12_H_21_O_4_), and 199 (C_11_H_19_O_3_), allowed to locate hydroxyl groups in the head (precisely at C9 and C10 positions) or in the tail of oxylipin (precisely at C12, C13, C15 and C16), respectively [19]. In particular, the MS/MS spectrum of compounds **21**, **22**, **24** and **25** showed diagnostic fragments at *m*/*z* 171, corresponding to the shortened acyl chain having the carboxyl group as COO^−^ and an aldehyde as terminal group originating from the rearrangement of the hydroxyl function on C9 following the cleavage of the C9–C10 bond [24]. Moreover, along with the molecular formula and the Ring Double Bond equivalent value (RDB), the fragmentation pattern allowed in some cases to establish the double bond position.

All this diagnostic information permitted us to establish the structure of compounds **21** ([M − H]^−^ at *m*/*z* 327.2171) and **22** ([M − H]^−^ at *m*/*z* 329.2326) differing for 2 Da but showing the same product ion at nominal *m*/*z* 229. This was formed by neutral loss of 98 Da (for compound **21**) and 100 Da (for compound **22**) from the corresponding molecular ion by rearrangement of the acyl chain end-part and breakdown of the C12–C13 bond, highlighting the presence of an additional double bond in the end-part of compound **21** in comparison with **22**. 

By these considerations, compounds **21**–**25** could be putatively defined as reported in Table 2.

#### 2.3.2. Identification of Galactolipids (**26**–**32**, **34** and **36**)

The analysis of the LC-ESI/LTQOrbitrap/MS profile highlighted the presence of pseudomolecular ions characterized by a molecular formula and a fragmentation pattern corresponding to glycolipids [25]. The LC-ESI HR/MS profile of *P. lucuma* pulp *n*-BuOH extract highlighted pairs of peaks—**26** and **27**, **28** and **29**, **30** and **32**—corresponding to the same molecular formula. The three pairs differed for the unsaturation degree. 

**Figure 3:**
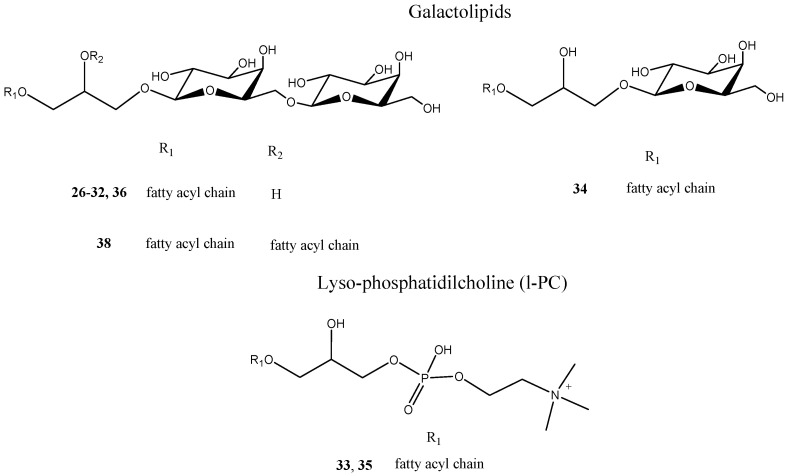
Polar lipids (galactolipids and Lyso-phosphatidilcholine (l-PC) isolated from *P. lucuma* pulp *n*-BuOH extract.

In the MS/MS spectrum of **26**–**30** and **32,** the same product ions at *m*/*z* 415, 397, and 235, leading to the assumption that **26**–**30** and **32** had a common structural backbone could be observed. The product ions at *m*/*z* 397 and 415 derived from the [M − H]^−^ ion by neutral losses differing by a water molecule, respectively, corresponding to 278 and 260 Da for **26** and **27**, to 280 and 262 Da for **28** and **29** and to 282 and 264 Da for **30** and **32**. They could be referred to [(M-RCOOH) − H]^−^ and [(M-R=CO) − H]^−^ ions, as confirmed by the presence of the RCOO fatty acyl ion at *m*/*z* 277, 279 and 281, respectively. The common product ion at *m*/*z* 235 originated from the consecutive neutral losses of a fatty acid unit and one hexose moiety (162 Da), and could be assigned to the glycosylated form of a mono-dehydrated glycerol backbone. According to the literature data and their molecular formulae [23], compounds **26**–**30**, **31** and **32** could be tentatively identified as digalactosylmonoacylglycerol (DGMG) species differing in the unsaturation degree and/or regiospecificity of the fatty acyl group (Figure 3).

Moreover, the analysis of LC-MS/MS data allowed to assign compound **31** as a digalactosylmonoacylglycerols. Analogously, compound **34** could be identified as a monogalactosylmonoacylglycerols (MGMG), which was glycosylated with only one sugar unit (Figure 3).

#### 2.3.3. Identification of Phosphatidylcholine Derivatives (**33** and **35**)

Analysis of the LC-ESI/LTQOrbitrap/MS profile in negative ionization mode of the pulp *n*-BuOH extract showed the presence of pseudomolecular ions corresponding to a molecular formula containing phosphorus heteroatom (Table 2 and Table S1). The fragmentation pattern of these ions showed diagnostic fragments of the polar heads attributable to the class of phosphatidylcholine derivatives [23]. For both compounds **33** and **35**, in agreement with the literature data, the formation in negative ion mode of [(M + FA) − H]^−^ formic acid adducts for phosphatidylcholine derivatives was evident. Moreover, in tandem mass spectra of compounds **33** and **35,** a product ion formed by the neutral loss of 60 Da from [(M + FA) − H]^−^ was observed, which allowed to identify this compound as l-PC (lyso-form of phosphatidylcholine) (Figure 3). This product ion could be attributed to the [(M-15) − H]^−^ ion, in which the l-PC derivative lost a methyl group from the choline head group to generate formic acid methyl ester [15].

### 2.4. LC-MS Analysis of P. lucuma Skin MeOH Extract

A preliminary metabolite fingerprint of *P. lucuma* skin MeOH extract was obtained by HR-LC-ESI-Orbitrap-MS analysis in negative ion mode. The LC-MS profile (Figure 1) showed eight main ion peaks which, on the basis of molecular formula, mass fragmentation behavior, and the literature data, could be defined as phenolics, distinguishable into galloyl 1-*O*-glucopyranoside (**1**), ellagic acid (**37**), flavonoids (**15**, **17** and **19**), as well as polar lipids characterized by the presence of oxylipins (**21** and **22**) and a galactolipid (**38**) (Table 2 and Table S1). Among these metabolites, compounds **37** and **38** were not detected in the HR-LC-ESI-Orbitrap-MS profile of the pulp extract. Compound **37** showed a precursor ion [M − H]^−^ at *m*/*z* 300.0253, corresponding to the molecular formula C_15_H_8_O_7_ attributed to ellagic acid, previously reported in lucuma [3,5]. As evident in the HR-LC-ESI-Orbitrap-MS profile of *P. lucuma* skin MeOH extract, reported in Figure 1, the main compound is represented by taxifolin (**15**) and in smaller extent by eriodictyol (**17**).

Moreover, the analysis of LC-MS/MS data allowed to assign compound **38** as digalactosyldiacylglycerols (DGDG) [25], by considering the presence in its spectra of the characteristic product ions at *m*/*z* 415 and 397, corresponding to the digalactosylglycerol anion in the whole and in monodehydrated form, respectively (Table 2).

### 2.5. Antioxidant Activity of Isolated Metabolites by P. lucuma 

The antioxidant capacity of isolated compounds was determined by TEAC assay (Table 3). Gallocatechin (**2**), epigallocatechin (**5**) and gallocatechin gallate (**11**) showed the strongest activity when compared with rutin, used as a reference compound. Taxifolin (**15**), the main compound occurring in the skin extract, showed a good activity with a value of 3.53 mM. This feature gives an interesting opportunity to valorize the skins for alternative uses, allowing companies to reduce their manufacturing costs.

## 3. Materials and Methods

### 3.1. Reagents 

HPLC-grade and extraction solvents were purchased by VWR International PBI (Milano, Italy). LC-MS grade solvents were purchased by Merck (Darmstadt, Germany). 2,2-diphenyl-1-picrylhydrazyl (DPPH^•^), 2,2′-azino-bis-(3-ethylbenzothiazoline-6-sulphonic acid (ABTS^•+^), potassium persulfate (K_2_S_2_O_8_), Folin-Ciocalteu reagent, Trolox, phosphate-buffered saline (PBS) solution, gallic acid and MeOH-d_4_ were purchased from Sigma-Aldrich (Darmstadt, Germany). 

### 3.2. Plant Material and Extraction

*Pouteria lucuma* fruits have been purchased on Fruttaweb. The skins have been removed by pulp manually and pulp was separated from the seeds. A voucher specimen has been deposited in this Department (n. 155).

*P. lucuma* pulps and skins were stored in the freezer at temperature −5 °C, after some days they were submitted to lyophilization to obtain 250 g and 71.65 g, respectively.

Pulps were extracted by maceration employing solvent with increasing polarity. A pre-extraction procedure was used to remove undesirable components, such as fatty acids, employing *n*-hexane (900 mL, three times for three days) and chloroform (900 mL, three times for three days). Afterward, extraction with methanol (900 mL, three times for three days) was performed. After filtration and evaporation of the solvent to dryness in vacuo 35.41 g of MeOH crude extracts were obtained. The analysis carried out by TLC (Thin Layer Chromatography) showed an important presence of sugars; thus, two repartitions with *n*-BuOH and water (1:1) were carried out. 19.19 g of extract were used, solubilized in 200 mL of water and 200 mL of butanol, obtaining 18.40 mg of the water component and 0.73 mg of the BuOH component.

*P. lucuma* skins (71.65 g) were extracted in the same way by maceration employing *n*-hexane (155 mL, three times for three days), chloroform (155 mL, three times for three days), and methanol (155 mL, three times for three days). After filtration and evaporation of the solvent to dryness in vacuo, 240 mg of MeOH crude extract was obtained.

### 3.3. Total Phenolic Content

The total phenolic content of the extracts was determined by Folin-Ciocalteu assay following the procedure previously reported with slight modifications [12]. 

The extracts of pulp and skin have been dissolved in methanol to reach a concentration of 0.5 mg/mL. In the centrifuge tubes, Folin-Ciocalteu phenol reagent (0.5 mL), extract (0.5 mL), saturated sodium carbonate solution (1 mL) have been mixed and the volume has been adjusted with distilled water to 10 mL. The mixtures were allowed to react at room temperature for 45 min (until the characteristic blue color developed) and then centrifuged at 1046 RCF for 5 min. Absorbance of the clear supernatant has been measured at 517 nm on a UV-visible spectrophotometer (Thermo Scientific Multiskan Go, Waltham, MA, USA). A control without FC reagent and a blank with methanol instead of sample have been included in the assay.

A calibration curve of the standard gallic acid was used, following the same procedure used for the extracts. For gallic acid, the calibration equation was was y = 0.0027x + 0.0982 (R^2^ = 0.993). All the experiments were performed in triplicate, and results were expressed as a mean of gallic acid equivalents (GAE mg/g dried extract).

### 3.4. DPPH^•^ Radical Scavenging Activity

DPPH^•^ radical scavenging activity of the extracts was evaluated by employing the protocol previously described [26] with slight modifications. In brief, stock solutions (10 mg/mL) of the different typologies of the extracts were used to obtain six different concentrations: from 10 to 500 μg/mL of pulp and skin extracts. The samples were stirred vigorously for 10 s and kept in the dark for 30 min; subsequently, the samples were put in a multiwall plate, in detail in each well has been added 244 µL of DPPH and 6 µL of each sample. The absorbance was measured at 517 nm using a Thermo Scientific Multiskan Go Spectrophotometer. A control solution was prepared by replacing the tested extracts with MeOH. The percentage of DPPH^•^ radical scavenging activity of the extracts was calculated as follows:

DPPH^•^ free radical scavenging activity (I%) = [(A0 − A)/A0] × 100. Where A0 is the absorbance of the control solution, and A is the absorbance of the DPPH^•^solution containing the extract. The percentage of DPPH^•^ radical scavenging activity (%) was plotted against the extract concentration (μg/mL) to determine the IC50. All the experiments were performed in triplicate.

### 3.5. ABTS^•+^ Radical Scavenging Activity

ABTS^•+^ radical scavenging activity was evaluated by using the method previously reported [17] with slight modifications. The radical ABTS^•+^ has been generated chemically by the oxidation of ABTS 2 mM (50 mL) with K_2_S_2_O_8_ 70 mM (0.2 L) after for 16 h of incubation in the dark at room temperature. Then, the mixture has been diluted with a phosphate-buffered saline (PBS) solution (pH = 7.4) to an absorbance of 0.700 ± 0.020 measured at 734 nm. 

30 μL of four concentrations of the extracts (250, 500, 750, 1000 μg/mL) were added to 300 µL of ABTS^•+^ radical solution in each well and their absorbance was measured at 734 nm by employing Thermo Scientific Multiskan Go Spectrophotometer. A negative control was prepared by using MeOH instead of the extract. Trolox was used as a reference standard, and results were expressed as TEAC value [17]. Each determination was performed in triplicate.

### 3.6. HR-LC-ESI-Orbitrap-MS and HR-LC-ESI-Orbitrap-MS/MS Analysis

HR-LC-ESI-Orbitrap-MS/MS analyses were performed in negative ion mode, employing a Thermo Scientific Accela HPLC System (Thermo Scientific, Dreieich, Germany) equipped with a Phenomenex (Torrance, CA, USA) Kinetex EVO C-18 column (150 × 2.10 mm, 5 µm), coupled to a LTQ-Orbitrap XL mass spectrometer (Thermo Fisher Scientific, San Jose, CA, USA) [15].

The employed mobile phases were water-formic acid (A, 99.9:0.1, *v*/*v*) and acetonitrile-formic acid (B, 99.9:0.1, *v*/*v*). The gradient conditions used for LC separation were the following: 0 min 5% B, 25 min 80% B (held for 3 min), 35 min 5% B (held for 10 min). The flow rate was 0.2 mL/min, injection volume was 4 µL (0.5 mg/mL) and the column was kept at room temperature.

The Orbitrap mass analyzer was calibrated according to the manufacturer’s directions [25]. The scan was collected in the Orbitrap at a resolution of 30,000 in a *m*/*z* range of 150–2000 Da. The *m*/*z* of each identified compound was calculated to 4 decimal places and measured with a mass accuracy < 3.88 ppm. The (−)ESI parameter settings were: capillary temperature at 280 °C, sheath gas flow at 15 (arbitrary units), auxiliary gas flow at 5 (arbitrary units), source voltage at 3.5 kV, capillary voltage at −48 V, and tube lens offset at −176.47 V. In LC-(−)ESI/HRMS experiments, the Total Ion Current (TIC) profile was produced by monitoring the intensity of all the ions produced and acquired in every scan during the chromatographic run. A normalized collision energy at 30%, a minimum signal threshold at 250, and an isolation width at 2.0 were used. MS/MS data were acquired by using the Data-Dependent Scan experiment, allowing to select the precursor ion as the most intense peak during LC-MS analyses. Xcalibur software (version 2.1, Dreieich, Germany) was used for instrument control, data acquisition and data analysis.

### 3.7. Isolation of Secondary Metabolites from P. lucuma Pulp Extract

*n*-BuOH extract of *P. lucuma* pulp was chromatographed by an Agilent 1260 Infinity system (Agilent Technologies, Palo Alto, CA, USA), equipped with a binary pump (G-1312C) and a UV detector (G-1314B), Phenomenex C18 Synergi-Hydro-RP (250 × 10 mm; 10 μm) column and a Rheodyne injector were used.

The sample was solubilized in methanol to obtain a 15 mg /100 μL and 100 μL were injected for a total of 14 chromatographic runs.

The following solvents were used: solvent (A) water-formic acid (0.1%) and (Bacetonitrile-formic acid (0.1%), with a flow of 2 mL/min, at a wavelength of 310. For all the chromatographic runs the following gradient was used: the first step involved the use of a 5% of B maintained for 5 min, in 45 min 80 % of B was reached, in 10 min it arrived at 100% of B, the latter % was held for 10 min.

In this way the following compounds **1** (1.8 mg, t_R_ = 5.2 min), **2** (2.1 mg, t_R_ = 8.6 min), **3** (2.2 mg, t_R_ = 23.0 min), **4** (2.0 mg, t_R_ = 24.5 min), **5** (1.2 mg, t_R_ = 8.8 min), **6** (1.7 mg, t_R_ R = 18.5 min), **7** (2.1 mg, t_R_ = 12.2 min), **8** (1.8 mg, t_R_ = 19.2 min), **9** (1.9 mg, t_R_ = 23.5 min), **10** (1.9 mg, t_R_ = 12.6 min), **11** (1.6 mg, t_R_ = 6.5 min), **12** (2.8 mg, t_R_ = 16.5 min), **13** (1.6 mg, t_R_ = 17.8 min), **14** (2.6 mg, t_R_ = 19.6 min), **15** (3.1 mg, t_R_ = 20.8 min), **16** (1.3 mg, t_R_ = 21.2 min), **17** (2.0 mg, t_R_ = 24.6 min), **18** (2.3 mg, t_R_ = 24.0 min), **19** (1.2 mg, t_R_ = 23.3 min), and **20** (2.3 mg, t_R_ = 29.5 min) were isolated. The structures of these compounds have been unambiguously elucidated by NMR research.

### 3.8. NMR Analysis

NMR spectroscopic data were acquired in MeOH-*d*_4_ (99.95%, Sigma-Aldrich) on a Bruker DRX-600 spectrometer (Bruker BioSpin GmBH, Rheinstetten, Germany) equipped with a Bruker 5 mm TCI CryoProbe at 300 K. Data processing was carried out with Topspin 3.2 software (Bruker BioSpin, Rheinstetten, Germany) Standard pulse sequences and phase cycling were used for DQF-COSY, HSQC, HMBC, and ROESY spectra.

## 4. Conclusions

In the present study, a total of 36 compounds were detected in *P. lucuma* pulp *n*-BuOH extract using a LC-ESI/LTQOrbitrap/MS/MS analysis. The phenolic compounds were isolated and their structures were unambiguously identified by NMR experiments. So far, the fruits have been studied mainly for their content of sugars, organic acids, total phenolics and total carotenoids and this is the first accurate investigation highlighting the structures of the compounds occurring in the extract.

A large number of papers on food plant metabolomics often describing incomplete characterization of plant constituents or tentative structures have been reported. It is evident that the putative attribution of structures by the only MS analysis should be confirmed by isolation of compounds and structure elucidation by NMR experiments. In this work, a strategy based on the combination of LC-MS and NMR techniques has been used as a powerful tool to achieve a deeper knowledge of lucuma metabolite profile.

Some of the isolated compounds showed a radical scavenging activity higher than rutin, used as a reference compound. On the basis of the obtained results, lucuma might be considered as a rich source of bioactives with antioxidant properties.

Analysis of the LC-ESI/LTQOrbitrap profile, accurate mass measurements, fragmentation pattern analyses, and comparison with literature data allowed to putatively identify 16 lipid compounds belonging to oxylipin, glycolipid and phospholipid classes, all of them for the first time described in lucuma. It is noteworthy that the extract contained a wide range of polar lipids, along with phenolic compounds. Considering the biological activities reported for lipid classes and their effects on human health [25], these data reinforce the use of lucuma in human nutrition as a food rich in different classes of bioactive and healthy lipids with beneficial effects.

To encourage the recycling and exploitation of lucuma by-products, preliminarily the total phenolic content of the skin extract was evaluated (560.69 mg GAE/g extract). In order to correlate the total phenolic content of the extract to its chemical composition, a LC-ESI/LTQOrbitrap/MS/MS analysis was carried out on *P. lucuma* skin extract. Taxifolin (**15**) represents the predominant compound along with eriodictyol (**17**). Moreover, taxifolin (**15**) showed a strong radical scavenging activity with a TEAC value of 3.53. This work demonstrated that lucuma skins, which represent an industry waste from lucuma processing could be useful for the preparation of food supplements.

## Figures and Tables

**Table 1:** Total phenolic content (TPC), DPPH^•^ and ABTS^•+^ radical scavenging activity of the extracts of *P. lucuma* pulp and skin evaluated by spectrophotometric assays.

*P. lucuma*	TPC ^a^	DPPH^•b^	ABTS^•+c^
MeOH extract of skin *	560.69 ± 4.76	52.71 ± 1.47 *	3.67 ± 0.27
*n*-BuOH extract of pulp **	93.53 ± 4.83	150.00 ± 2.55 **	2.24 ± 0.12
Vitamin C		4.85 ± 0.05	-
rutin (mM)			4.65 ± 0.15

* Range concentration 10–200 (μg/mL); ** Range concentration 50–500 (μg/mL). ^a^ Values are expressed as gallic acid equivalents (GAE) mg/g of dried extract. ^b^ Values are expressed as IC_50_ in μg/mL. ^c^ Values are expressed as concentration (mM) of a standard Trolox solution exerting the same antioxidant activity of a 1 mg/mL solution of the tested extract. Results are expressed as mean of three experiments.

**Table 2:** Compounds identified in *P. lucuma* pulp *n*-BuOH extract and *P. lucuma* skin MeOH extract by LCESI/LTQOrbitrap/MS/MS (negative ion mode).

MeOH Extract of *P. lucuma* Pulp								
	Compound	t_R_ (min)	Molecular Formula	[(M + HCOOH) − H]^−^	[M − H]^−^	Δ ppm	Product Ions	Classification
**1**	galloyl 1-*O*-glucopyranoside	1.51	C_13_H_16_O_10_		331.0664	1.35	169	phenolic
**2**	gallocatechin	4.84	C_15_H_14_O_7_		305.06561	1.61	287, 261, 221, 175, 125	flavanol
**3**	*p*-coumaric acid	5.60	C_9_H_8_O_3_	209.0451		2.97	145, 119	phenolic
**4**	*p*-ferulic acid	5.66	C_10_H_10_O_4_	239.0554		1.78	149, 133	phenolic
**5**	epigallocatechin	6.28	C_15_H_14_O_7_		305.0659	0.34	287, 261, 221, 175, 125	flavanol
**6**	*p*-coumaroyl 4-*O*-β-d-glucopyranoside	6.33	C_15_H_18_O_8_		325.0928	0.98	163, 145	phenolic
**7**	catechin	6.53	C_15_H_14_O_6_		289.0713	1.96	179, 151, 137	flavanol
**8**	*p*-feruloyl-4-*O*-β-d-glucopyranoside	6.65	C_16_H_20_O_9_		355.1025	0.48	193, 175	phenolic
**9**	4-hydroxybenzoic acid 4-*O*-β-d-glucopyranoside	6.96	C_13_H_16_O_8_		299.0763	0.56	137	phenolic
**10**	epicatechin	7.33	C_15_H_14_O_6_		289.0715	2.01	179, 151, 137	flavanol
**11**	gallocatechin-gallate	7.64	C_22_H_18_O_11_		457.0769	0.75	287, 169	flavanol
**12**	ampelopsin	7.89	C_15_H_12_O_8_		319.0456	2.24	301, 193	dihydroflavonol
**13**	myricetin 3-*O*-α-l-rhamnopyranoside	8.38	C_21_H_20_O_12_		479.0824	0.72	317	flavonol
**14**	resveratrol-3-*O*-β-d-glucopyranoside	8.87	C_20_H_22_O_8_		389.1232	0.14	227	phenolic
**15**	taxifolin	9.55	C_15_H_12_O_7_		303.0503	1.16	285, 177, 125	dihydroflavonol
**16**	quercetin 3-*O*-β-d-rhamnopyranoside	10.41	C_21_H_20_O_11_		447.0927	−0.20	301	flavonol glycoside
**17**	eriodictyol	10.91	C_15_H_12_O_6_		287.0550	0.02	179, 163, 153	dihydroflavonol
**18**	*p*-hydroxy benzoic acid	11.53	C_7_H_6_O_3_		137.0218	3.10	93	phenolic
**19**	quercetin	11.70	C_15_H_10_O_7_		301.0350	2.46	179, 151	flavonol
**20**	salycilic acid	11.83	C_7_H_6_O_3_		137.0218	3.10	93	phenolic
**21**	TriHoDe	13.01	C_18_H_32_O_5_		327.2171	0.51	309, 291, 273, 229, 221, 171	oxylipin
**22**	TriHoMe	13.69	C_18_H_34_O_5_		329.2326	0.32	311, 293, 229, 211, 199, 171	oxylipin
**23**	TriHoDe	14.92	C_18_H_28_O_4_		307.1909	0.48	289, 271, 243, 235, 209	oxylipin
**24**	hydroxy-epoxy-octadecadienoic acid	16.34	C_18_H_30_O_4_		309.2065	0.50	291, 273, 201, 171	oxylipin
**25**	hydroxy-epoxy-octadecadienoic acid isomer	16.71	C_18_H_30_O_4_		309.2065	0.5	291, 273, 201, 171	oxylipin
**26**	DGMG (18:3)	17.32	C_33_H_56_O_14_	721.3634		−2.54	675, 415, 397, 305, 277, 235, 205	galactolipid
**27**	DGMG (18:3)	17.69	C_33_H_56_O_146_	721.3631		−2.98	675, 415, 397, 305, 277, 235, 205	galactolipid
**28**	DGMG (18:2)	18.30	C_33_H_58_O_14_	723.3796		−3.47	677, 415, 397, 279, 235	galactolipid
**29**	DGMG (18:2)	18.74	C_33_H_58_O_14_	723.3795		−3.47	677, 415, 397, 279, 235	galactolipid
**30**	DGMG (18:1)	19.17	C_33_H_60_O_14_	725.3937		−3.87	679, 415, 397, 281, 235	galactolipid
**31**	DGMG (16:0)	19.42	C_31_H_58_O_14_	699.3788		−1.40	415, 397, 235	galactolipid
**32**	DGMG (18:1)	19.60	C_33_H_60_O_14_	725.3937		−3.87	679, 415, 397, 281, 235	galactolipid
**33**	l-PC (16:0)	19.98	C_24_H_50_O_7_NP	540.3296		0.028	480, 255	phosphatidylcholine
**34**	MGMG (16:3)	20.59	C_25_H_42_O_9_		485.2744	−0.27	235	galactolipid
**35**	l-PC (18:1)	20.59	C_26_H_52_O_7_NP	566.3451		−0.17	506, 281	phosphatidylcholine
**36**	DGMG (18:0)	21.70	C_33_H_62_O_14_	727.4096		−1.98	681, 397	galactolipid
MeOH extract of *P. lucuma* skin								
**1**	galloyl 1-*O*-glucopyranoside	1.51	C_13_H_16_O_10_		331.0668	1.39	169	phenolic
**15**	taxifolin	9.46	C_15_H_12_O_7_		303.0503	1.16	285, 177, 125	dihydroflavonol
**17**	eriodictyol	10.87	C_15_H_12_O_6_		287.0558	2.67	179, 163, 153	dihydroflavonol
**19**	quercetin	12.58	C_15_H_10_O_7_		301.0346	1.00	179, 151	flavonol
**21**	TriHoDe	12.90	C_18_H_32_O_5_		327.2169	0.88	229, 211, 171	oxylipin
**22**	TriHOME	13.83	C_18_H_34_O_5_		329.2326	1.18	293, 229, 211, 199, 171	oxylipin
**37**	ellagic acid	14.69	C_14_H_6_O_8_		300.9983	−3.85	257, 201	phenolic
**38**	DGDG (18.3, 16:0)	30.50	C_49_H_86_O_15_		959.5934	−0.35	913, 415, 397, 277, 255	galactolipid

**Table 3:** ABTS^•+^ radical scavenging activity of isolated compounds **1**–**20**.

	Compound	TEAC Value (mM ± SD)
**1**	galloyl 1-*O*-glucopyranoside	3.24 ± 0.07
**2**	gallocatechin	5.12 ± 0.21
**3**	*p*-coumaric acid	0.70 ± 0.03
**4**	*p*-ferulic acid	1.93 ± 0.01
**5**	epigallocatechin	5.29 ± 0.30
**6**	*p*-coumaroyl 4-*O*-β-d-glucopyranoside	0.79 ± 0.01
**7**	catechin	2.56 ± 0.27
**8**	*p*-feruloyl-4-*O*-β-d-glucopyranoside	2.01 ± 0.05
**9**	4-hydroxybenzoic acid 4-*O*-β-d-glucopyranoside	2.46 ± 0.07
**10**	epicatechin	2.14 ± 0.24
**11**	gallocatechin-gallate	5.92 ± 0.16
**12**	ampelopsin	2.29 ± 0.03
**13**	myricetina 3-*O*-α-l-rhamnopyranoside	4.41 ± 0.23
**14**	resveratrol-3-*O*-β-d-glucopyranoside	2.00 ± 0.40
**15**	taxifolin	3.53 ± 0.29
**16**	quercetin 3-*O*-β-d-rhamnopyroside	4.85 ± 0.10
**17**	eriodictyol	2.12 ± 0.06
**18**	*p*-hydroxy benzoic acid	2.25 ± 0.09
**19**	quercetin	4.71 ± 0.27
**20**	salycilic acid	2.12 ± 0.03
	rutin	4.65 ± 0.15

Results are expressed as mean of three experiments.

## Data Availability

The data presented in this study are available in supplementary materials.

## Supplementary Materials

The following materials are available online: Table S1. Molecular formula [M − H]^−^, [(M + HCOOH) − H]^−^, characteristic product ions (molecular formula and intensity) occurring in *P. lucuma pulp n*-BuOH extract and *P. lucuma skin* MeOH extract, identified by LCESI/LTQOrbitrap/MS/MS (negative ion mode); Figure S1. Chromatogram of *P. lucuma* pulp extract on the C18 Synergi-Hydro-RP column; Figures S2–S21. ^1^H NMR Spectrum (600 MHz, CD_3_OD) of compound **1**–**20**.

## Sample Availability

Samples of the compounds **1**–**20** are available from the authors.
