# Supplementary material for: Pouteria lucuma Pulp and Skin: In Depth Chemical Profile and Evaluation of Antioxidant Activity
Source: Molecules. 2021 Aug 29;26(17):5236. doi: 10.3390/molecules26175236 (PMC8434328; doi:10.3390/molecules26175236)
Supplement: Supplementary file 1 [file molecules-26-05236-s001.zip › molecules-1344676-supplementary.pdf]

*Supplementary Materials*

# *Pouteria lucuma* Pulp and Skin: In Depth Chemical Profile and Evaluation of Antioxidant Activity

**Milena Masullo[1], Antonietta Cerulli[1], Cosimo Pizza[1] and Sonia Piacente[1,*]**

1 Dipartimento di Farmacia, Università degli Studi di Salerno, via Giovanni Paolo II n. 132, 84084 Fisciano (SA), Italy; piacente@unisa.it
* Correspondence: piacente@unisa.it; Tel.: +39089969763

**Table S1.** Molecular formula [M-H]$^-$, [(M+HCOOH)-H]$^-$, characteristic product ions (molecular formula and intensity) occurring in *P. lucuma* pulp *n*-BuOH extract and *P. lucuma* skin MeOH extract, identified by LCESI/LTQOrbitrap/MS/MS (negative ion mode).

**Figure S1.** Chromatogram of *P. lucuma* pulp extract on the C18 Synergi-Hydro-RP column

**Figure S2.** $^1$H NMR Spectrum (600 MHz, $CD_3OD$) of compound **1**

**Figure S3.** $^1$H NMR Spectrum (600 MHz, $CD_3OD$) of compound **2**

**Figure S4.** $^1$H NMR Spectrum (600 MHz, $CD_3OD$) of compound **3**

**Figure S5.** $^1$H NMR Spectrum (600 MHz, $CD_3OD$) of compound **4**

**Figure S6.** $^1$H NMR Spectrum (600 MHz, $CD_3OD$) of compound **5**

**Figure S7.** $^1$H NMR Spectrum (600 MHz, $CD_3OD$) of compound **6**

**Figure S8.** $^1$H NMR Spectrum (600 MHz, $CD_3OD$) of compound **7**

**Figure S9.** $^1$H NMR Spectrum (600 MHz, $CD_3OD$) of compound **8**

**Figure S10.** $^1$H NMR Spectrum (600 MHz, $CD_3OD$) of compound **9**

**Figure S11.** $^1$H NMR Spectrum (600 MHz, $CD_3OD$) of compound **10**

**Figure S12.** $^1$H NMR Spectrum (600 MHz, $CD_3OD$) of compound **11**

**Figure S13.** $^1$H NMR Spectrum (600 MHz, $CD_3OD$) of compound **12**

**Figure S14.** $^1$H NMR Spectrum (600 MHz, $CD_3OD$) of compound **13**

**Figure S15.** $^{1}$H NMR Spectrum (600 MHz, $CD_3OD$) of compound **14**

**Figure S16.** $^{1}$H NMR Spectrum (600 MHz, $CD_3OD$) of compound **15**

**Figure S17.** $^{1}$H NMR Spectrum (600 MHz, $CD_3OD$) of compound **16**

**Figure S18.** $^{1}$H NMR Spectrum (600 MHz, $CD_3OD$) of compound **17**

**Figure S19.** $^{1}$H NMR Spectrum (600 MHz, $CD_3OD$) of compound **18**

**Figure S20.** $^{1}$H NMR Spectrum (600 MHz, $CD_3OD$) of compound **19**

**Figure S21.** $^{1}$H NMR Spectrum (600 MHz, $CD_3OD$) of compound **20**

**Table S1.** Molecular formula $[M-H]^-$, $[(M+HCOOH)-H]^-$, characteristic product ions (molecular formula and intensity) occurring in *P. lucuma* pulp *n*-BuOH extract and *P. lucuma* skin MeOH extract, identified by LCESI/LTQOrbitrap/MS/MS (negative ion mode).

| | **compound** | **Molecular formula** | **[(M+HCOOH)-H]⁻** | **[M-H]⁻** | **product ions (molecular formula, intensity)** |
|---|---|---|---|---|---|
| **1** | galloyl 1-*O*- glucopyranoside | $C_{13}H_{16}O_{10}$ | | 331.0664 | 169.03 ($C_7H_5O_5$, 99.6) |
| **2** | gallocatechin | $C_{15}H_{14}O_7$ | | 305.0656 | 287.16 ($C_{15}H_{11}O_6$, 33.2),<br>125.15 ($C_6H_5O_3$, 13.6) |
| **3** | *p*-coumaric acid | $C_9H_8O_3$ | 209.0451 | | 119.08 ($C_8H_7O_0$, 26.6), |
| **4** | *p*-ferulic acid | $C_{10}H_{10}O_4$ | 239.0554 | | 133.05 ($C_8H_5O_2$, 76.6), |
| **5** | epigallocatechin | $C_{15}H_{14}O_7$ | | 305.0659 | 287.01 ($C_{15}H_{11}O_6$, 36.8),<br>125.11 ($C_6H_5O_3$, 16.3) |
| **6** | *p*-coumaroyl 4-*O*-β-D-glucopyranoside | $C_{15}H_{18}O_8$ | | 325.0928 | 163.12 ($C_6H_7O_3$, 21.5), |
| **7** | catechin | $C_{15}H_{14}O_6$ | | 289.0713 | 179.05 ($C_9H_7O_4$, 17.47)<br>151.01($C_8H_7O_3$,0.75 )<br>137.08 ($C_7H_5O_3$,3.69 ) |
| **8** | *p*-feruloyl-4-*O*-β-D-glucopyranoside | $C_{16}H_{20}O_9$ | | 355.1025 | 193.18 ($C_{10}H_9O_4$, 36.8) |
| **9** | 4-hydroxybenzoic acid 4-*O*-β-D-glucopyranoside | $C_{13}H_{16}O_8$ | | 299.0763 | 137.02 ($C_7H_6O_3$, 65.9) |
| **10** | epicatechin | $C_{15}H_{14}O_6$ | | 289.0715 | 179.01 ($C_9H_7O_4$, 13.73)<br>151.02($C_8H_7O_3$, 0.93)<br>137.07 ($C_7H_5O_3$,3.68 ) |
| **11** | gallocatechin-gallate | $C_{22}H_{18}O_{11}$ | | 457.0769 | 287.11 ($C_{15}H_{11}O_6$, 32.21) |

| | | | | | |
|---|---|---|---|---|---|
| | | | | | 168.99 ($C_7H_5O_5$, 100.00) |
| **12** | ampelopsin | $C_{15}H_{12}O_8$ | | 319.0456 | 301.12 ($C_{15}H_9O_7$, 38.46) |
| **13** | myricetin 3-*O*-α-L-rhamnopyranoside | $C_{21}H_{20}O_{12}$ | | 479.0824 | 317.10 ($C_{15}H_9O_8$, 58.95) |
| **14** | resveratrol-3-*O*-β-D-glucopyranoside | $C_{20}H_{22}O_8$ | | 389.1232 | 227.15 ($C_{14}H_{11}O_3$, 99.85) |
| **15** | taxifolin | $C_{15}H_{12}O_7$ | | 303.0503 | 285.07 ($C_{15}H_9O_6$, 100.0),<br>176.96 ($C_9H_5O_4$, 10.87) |
| **16** | quercetin 3-*O*-β-D-rhamnopyranoside | $C_{21}H_{20}O_{11}$ | | 447.0927 | 300.99 ($C_{15}H_9O_7$, 66.06) |
| **17** | eriodictyol | $C_{15}H_{12}O_6$ | | 287.0550 | 179.21 ($C_9H_7O_4$, 56.06)<br>153.07 ($C_7H_5O_4$, 32.03) |
| **18** | *p*-hydroxy benzoic acid | $C_7H_6O_3$ | | 137.0218 | 93.05 ($C_6H_5O$, 65.01) |
| **19** | quercetin | $C_{15}H_{10}O_7$ | | 301.0350 | 179.01 ($C_8H_3O_5$, 100.00)<br>151.01 ($C_8H_7O_3$, 71.98) |
| **20** | salycilic acid | $C_7H_6O_3$ | | 137.0218 | 93.03 ($C_6H_5O$, 75.03) |
| **21** | TriHoDe | $C_{18}H_{32}O_5$ | | 327.2171 | 229.05 ($C_{12}H_{21}O_4$, 100.00),<br>171.03 ($C_9H_{15}O_3$, 52.18) |
| **22** | TriHoMe | $C_{18}H_{34}O_5$ | | 329.2326 | 229.07 ($C_{12}H_{21}O_4$, 100.00),<br>171.08 ($C_9H_{15}O_3$, 55.15) |
| **23** | TriHoDe | $C_{18}H_{28}O_4$ | | 307.1909 | 171.03 ($C_9H_{15}O_3$, 55.26) |
| **24** | hydroxy-epoxy-octadecadienoic acid | $C_{18}H_{30}O_4$ | | 309.2065 | 201.21 ($C_{10}H_{17}O_4$, 52.99)<br>171.04 ($C_9H_{15}O_3$, 67.88) |
| **25** | hydroxy-epoxy-octadecadienoic acid isomer | $C_{18}H_{30}O_4$ | | 309.2065 | 201.26 ($C_{10}H_{17}O_4$, 56.83)<br>171.02 ($C_9H_{15}O_3$, 67.85) |
| **26** | DGMG (18:3) | $C_{33}H_{56}O_{14}$ | 721.3634 | | 415.26 ($C_{15}H_{27}O_{13}$, 3.35)<br>397.21 ($C_{15}H_{25}O_{12}$, 18.30)<br>235.15 ($C_9H_{15}O_7$, 0.43) |
| **27** | DGMG (18:3) | $C_{33}H_{56}O_{146}$ | 721.3631 | | 415.29 ($C_{15}H_{27}O_{13}$,3.99)<br>397.24 ($C_{15}H_{25}O_{12}$, 15.78)<br>235.12 ($C_9H_{15}O_7$, 0.33) |
| **28** | DGMG (18:2) | $C_{33}H_{58}O_{14}$ | 723.3796 | | 415.00 ($C_{15}H_{27}O_{13}$, 3.56)<br>397.16 ($C_{15}H_{25}O_{12}$, 18.31) |
| **29** | DGMG (18:2) | $C_{33}H_{58}O_{14}$ | 723.3795 | | 415.26 ($C_{15}H_{27}O_{13}$, 0.99)<br>397.26 ($C_{15}H_{25}O_{12}$, 11.36) |
| **30** | DGMG (18:1) | $C_{33}H_{60}O_{14}$ | 725.3937 | | 415.52 ($C_{15}H_{27}O_{13}$, 1.18)<br>397.30 ($C_{15}H_{25}O_{12}$, 4.76) |
| **31** | DGMG (16:0) | $C_{31}H_{58}O_{14}$ | 699.3788 | | 415.03 ($C_{15}H_{27}O_{13}$, 0.58)<br>397.19 ($C_{15}H_{25}O_{12}$, 13.89)<br>235.14 ($C_9H_{15}O_7$, 0.62) |
| **32** | DGMG (18:1) | $C_{33}H_{60}O_{14}$ | 725.3937 | | 415.30 ($C_{15}H_{27}O_{13}$, 1.35)<br>397.49 ($C_{15}H_{25}O_{12}$, 0.68) |
| **33** | l-PC (16:0) | $C_{24}H_{50}O_7NP$ | 540.3296 | | 255.37 ($C_{16}H_{31}O_2$, 100) |

| | | | | | |
|---|---|---|---|---|---|
| **34** | MGMG (16:3) | $C_{25}H_{42}O_9$ | | 485.2744 | 235.14 ($C_9H_{15}O_7$, 0.35) |
| **35** | l-PC (18:1) | $C_{26}H_{52}O_7NP$ | 566.3451 | | 281.25 ($C_{18}H_{33}O_2$, 46.23) |
| **36** | DGMG (18:0) | $C_{33}H_{62}O_{14}$ | 727.4096 | | 415.37 ($C_{15}H_{27}O_{13}$, 2.38) |
| | | | | | 397.24 ($C_{15}H_{25}O_{12}$, 17.97) |
| | | | | | 235.23 ($C_9H_{15}O_7$, 0.73) |

| | | | | | |
|---|---|---|---|---|---|
| **1** | galloyl 1-*O*- glucopyranoside | $C_{13}H_{16}O_{10}$ | | 331.0668 | 169.03 ($C_7H_5O_5$, 99.8) |
| **15** | taxifolin | $C_{15}H_{12}O_7$ | | 303.0503 | 285.08 ($C_{15}H_9O_6$, 100.0), |
| | | | | | 176.96 ($C_9H_5O_4$, 10.83) |
| **17** | eriodictyol | $C_{15}H_{12}O_6$ | | 287.0558 | 179.21 ($C_9H_7O_4$, 56.06) |
| | | | | | 153.07 ($C_7H_5O_4$, 32.03) |
| **19** | quercetin | $C_{15}H_{10}O_7$ | | 301.0346 | 179.01 ($C_8H_3O_5$, 100.00) |
| | | | | | 151.02 ($C_8H_7O_3$, 71.96) |
| **21** | TriHoDe | $C_{18}H_{32}O_5$ | | 327.2169 | 229.04 ($C_{12}H_{21}O_4$, 100.00), |
| | | | | | 171.03 ($C_9H_{15}O_3$, 52.16) |
| **22** | TriHOME | $C_{18}H_{34}O_5$ | | 329.2326 | 229.16 ($C_{12}H_{21}O_4$, 100.00), |
| | | | | | 170.96 ($C_9H_{15}O_3$, 77.24) |
| **37** | ellagic acid | $C_{14}H_6O_8$ | | 300.9983 | 257.04 ($C_{13}H_5O_6$, 76.37) |
| **38** | DGDG (18.3, 16:0) | $C_{49}H_{86}O_{15}$ | | 959.5934 | 415.26 ($C_{15}H_{27}O_{13}$, 3.35) |
| | | | | | 397.21 ($C_{15}H_{25}O_{12}$, 18.30) |
| | | | | | 235.15 ($C_9H_{15}O_7$, 0.43) |

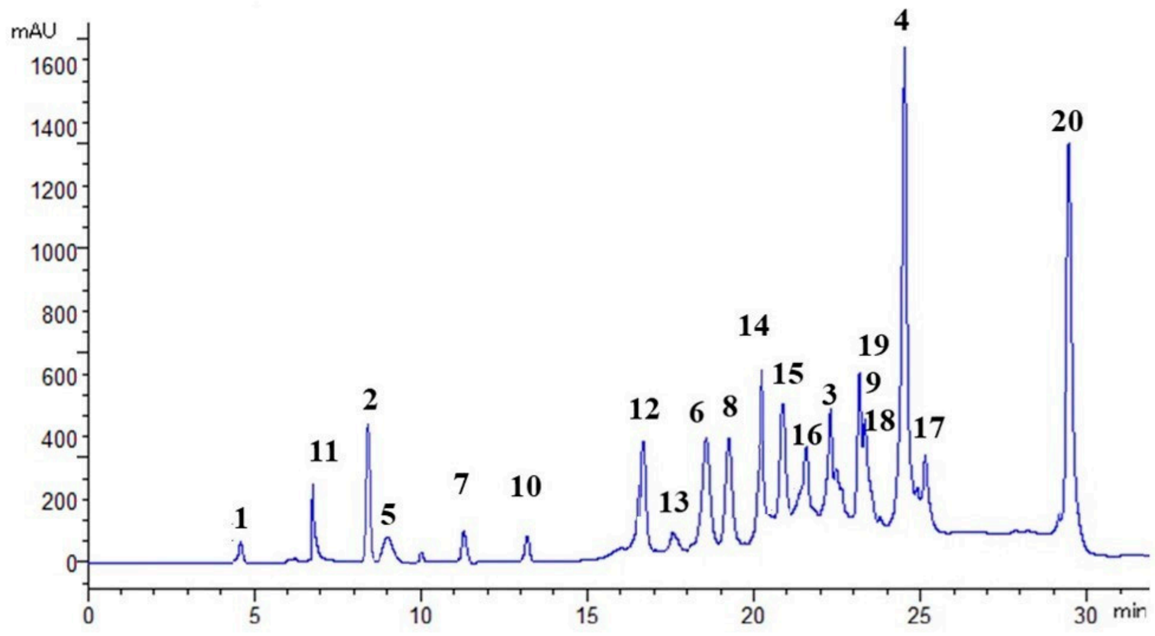


**Figure S1.** HPLC-UV Chromatogram of *P. lucuma* pulp extract at wavelength 310 nm.

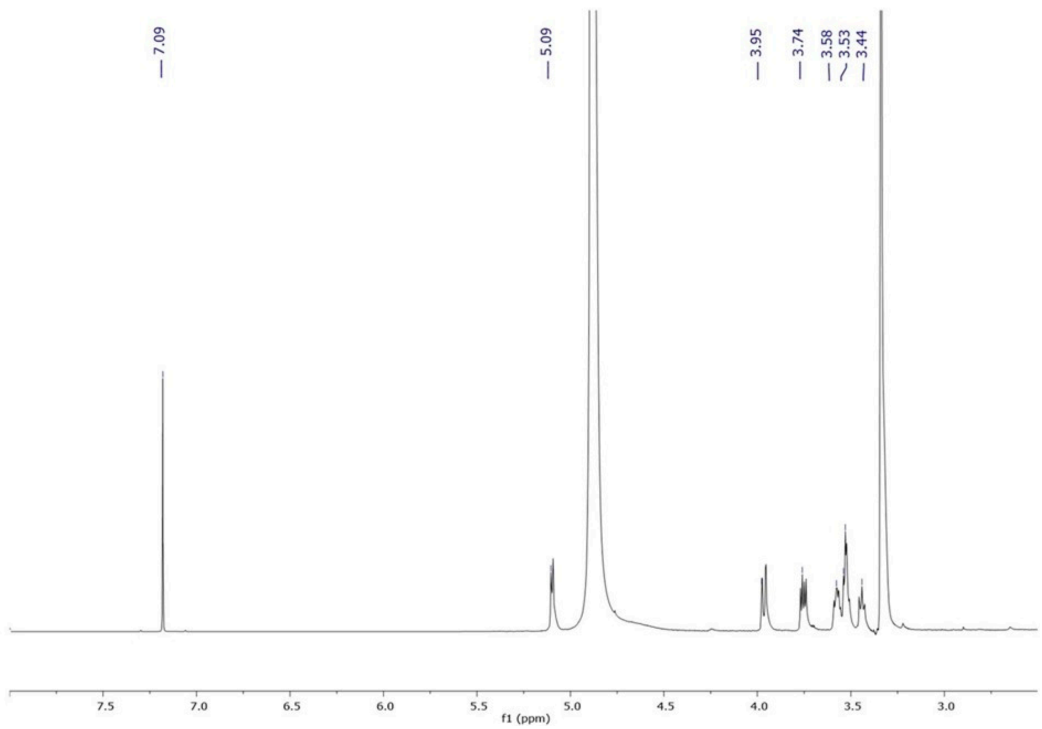


**Figure S2.** $^{1}$H NMR Spectrum (600 MHz, $CD_3OD$) of compound **1**

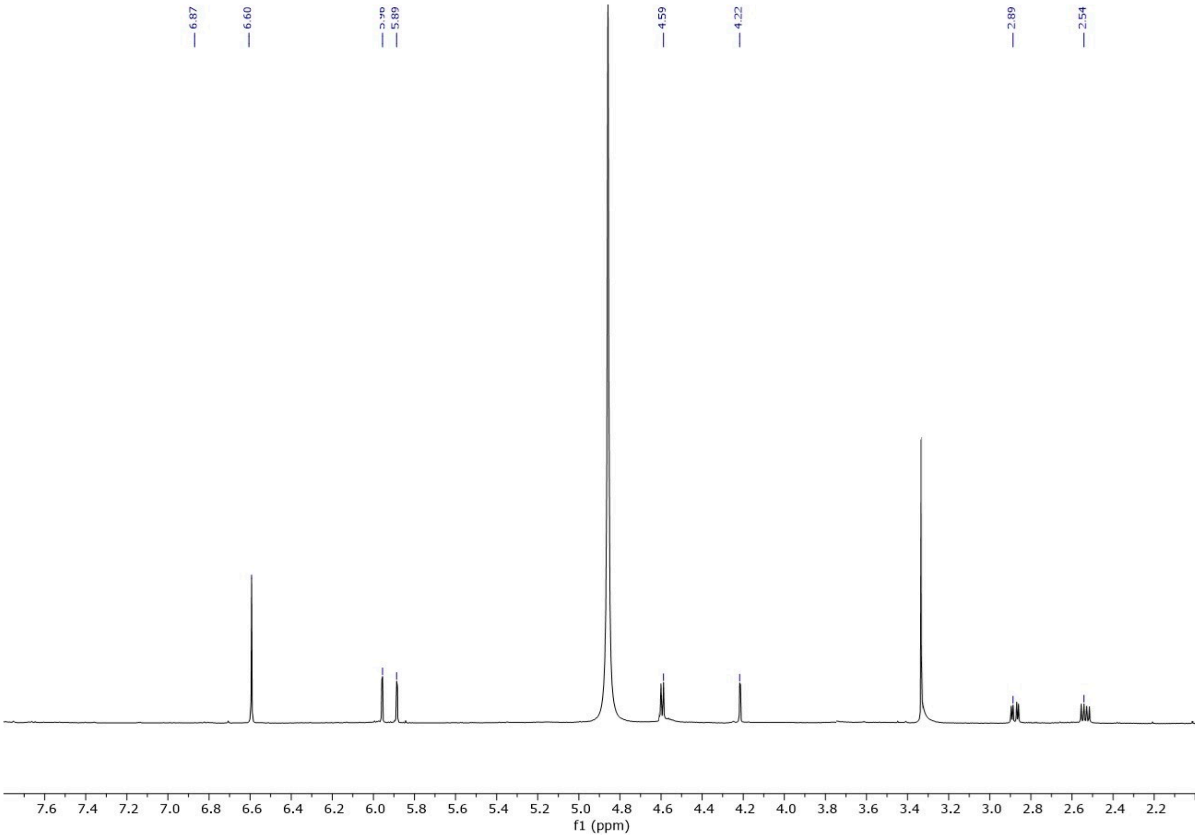


**Figure S3.** $^{1}$H NMR Spectrum (600 MHz, $CD_3OD$) of compound **2**

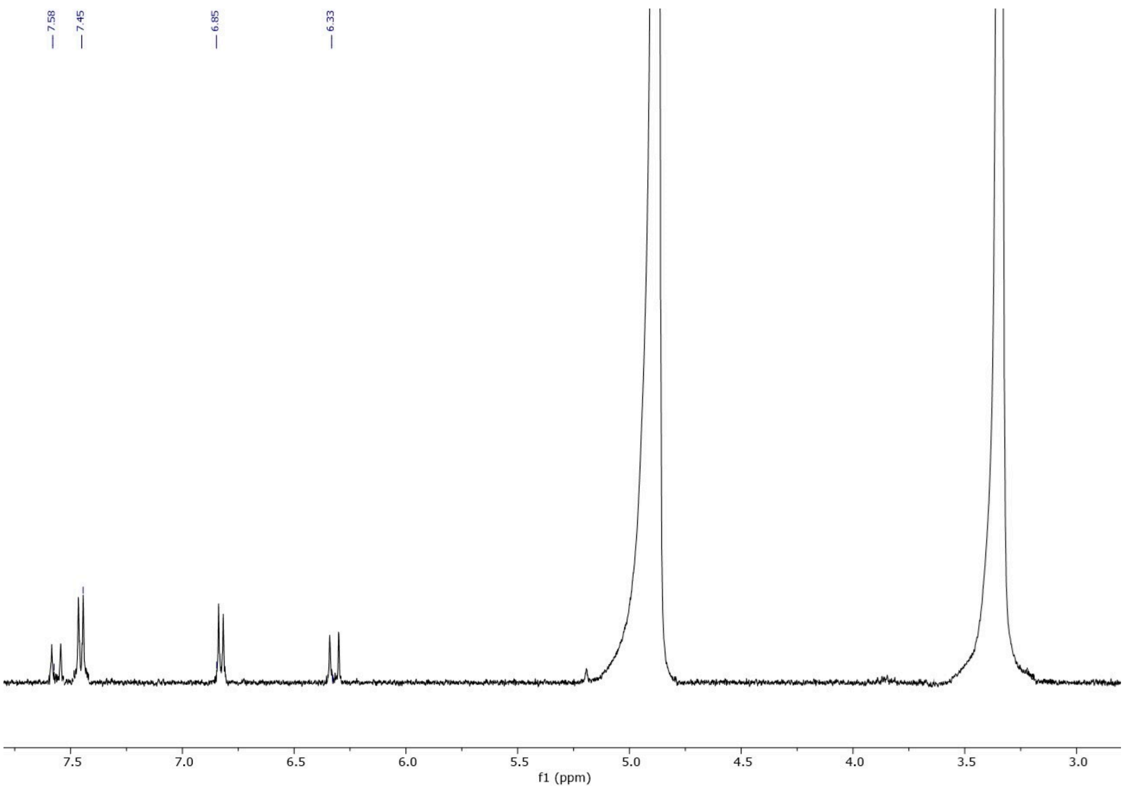


**Figure S4.** $^{1}H$ NMR Spectrum (600 MHz, $CD_3OD$) of compound **3.**

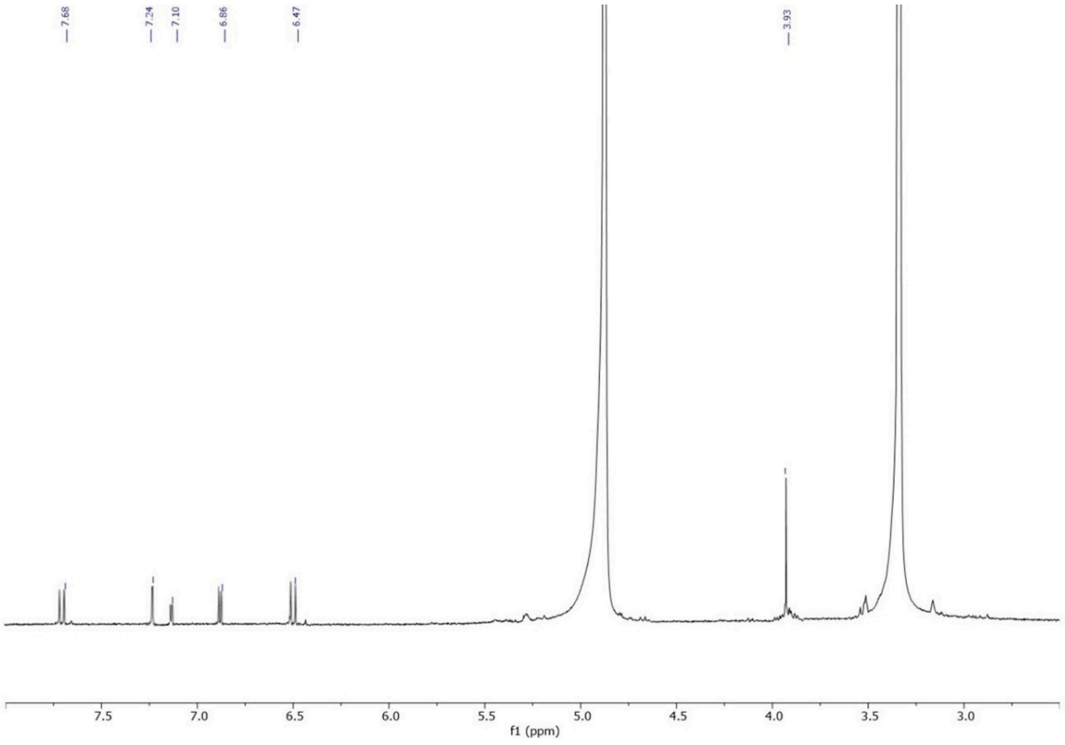


**Figure S5.** $^{1}H$ NMR Spectrum (600 MHz, $CD_3OD$) of compound **4.**

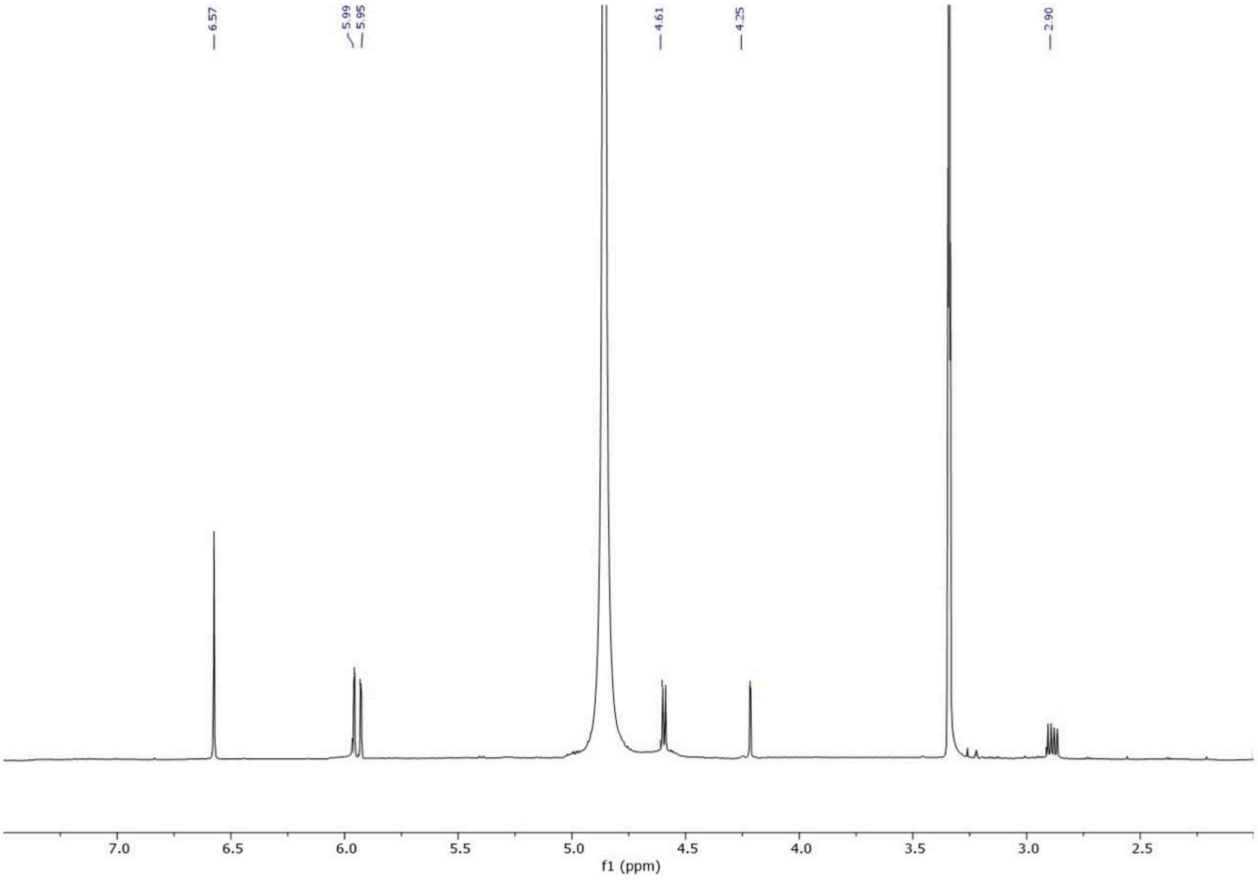


**Figure S6.** $^{1}$H NMR Spectrum (600 MHz, $CD_3OD$) of compound **5.**

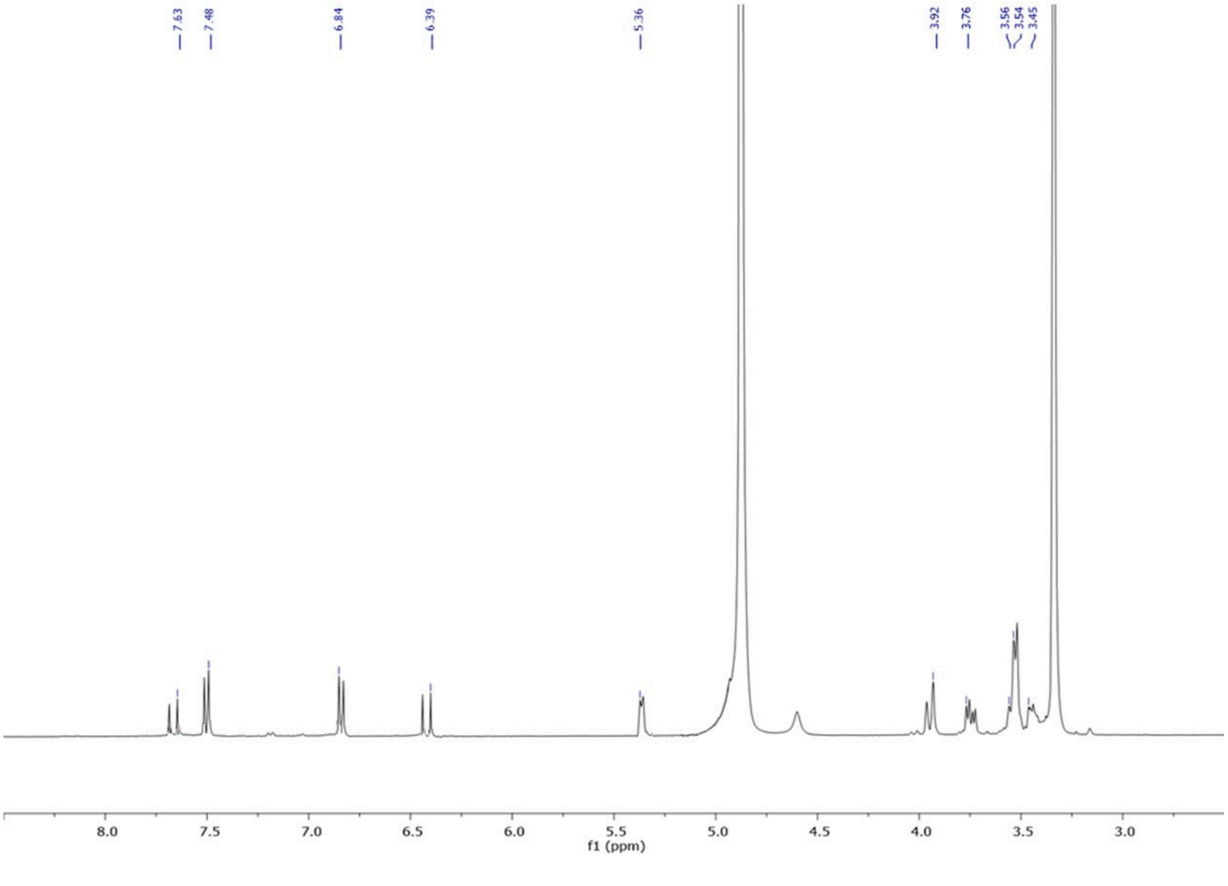

**Figure S7.** $^{1}$H NMR Spectrum (600 MHz, $CD_3OD$) of compound **6.**

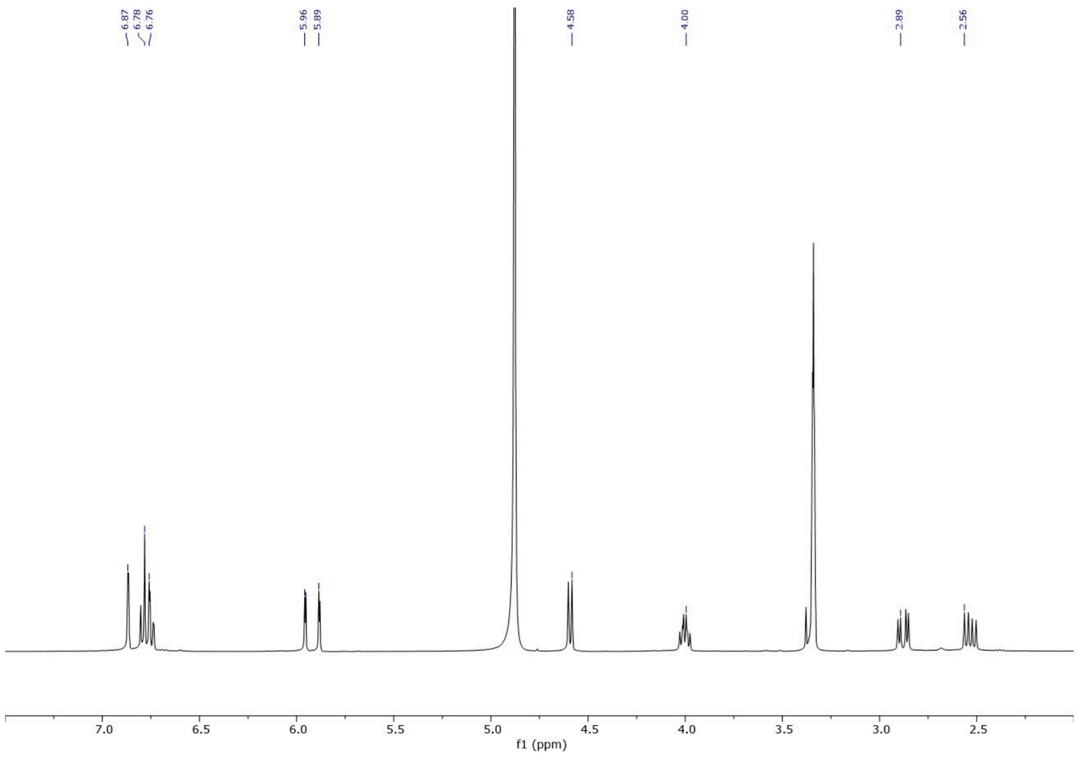


**Figure S8.** $^{1}$H NMR Spectrum (600 MHz, $CD_3OD$) of compound **7.**

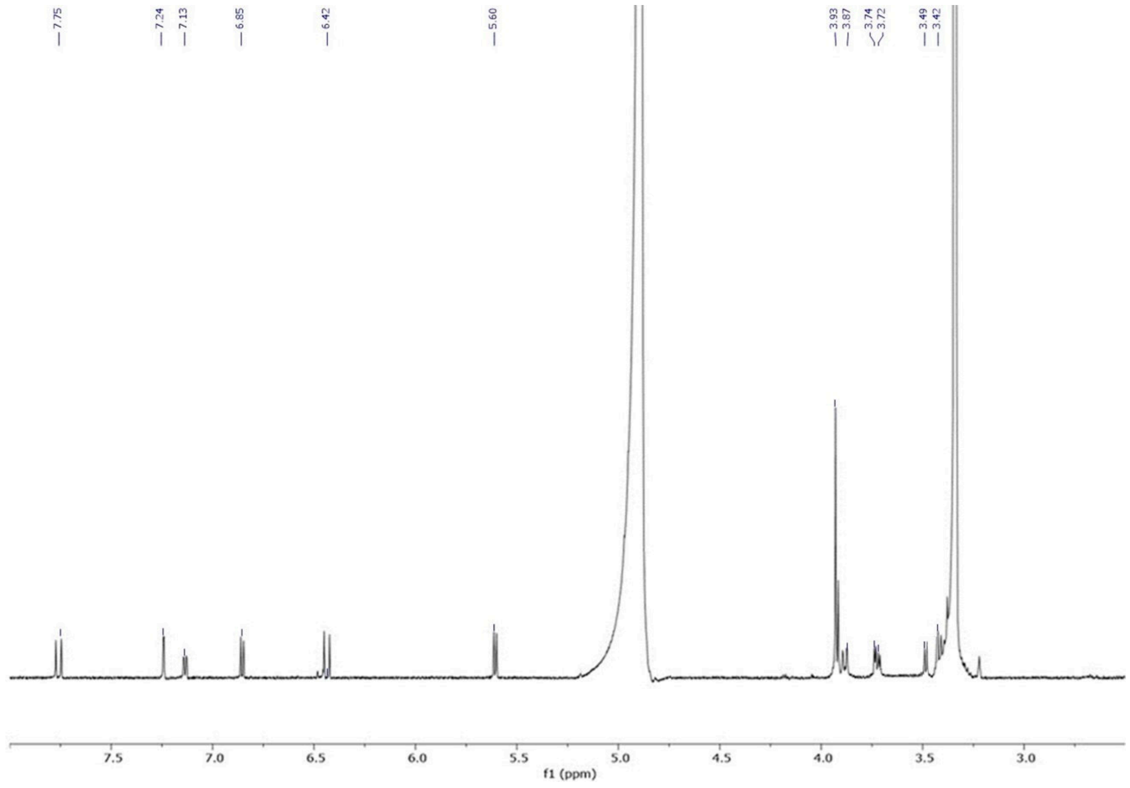


**Figure S9.** $^{1}$H NMR Spectrum (600 MHz, $CD_3OD$) of compound **8.**

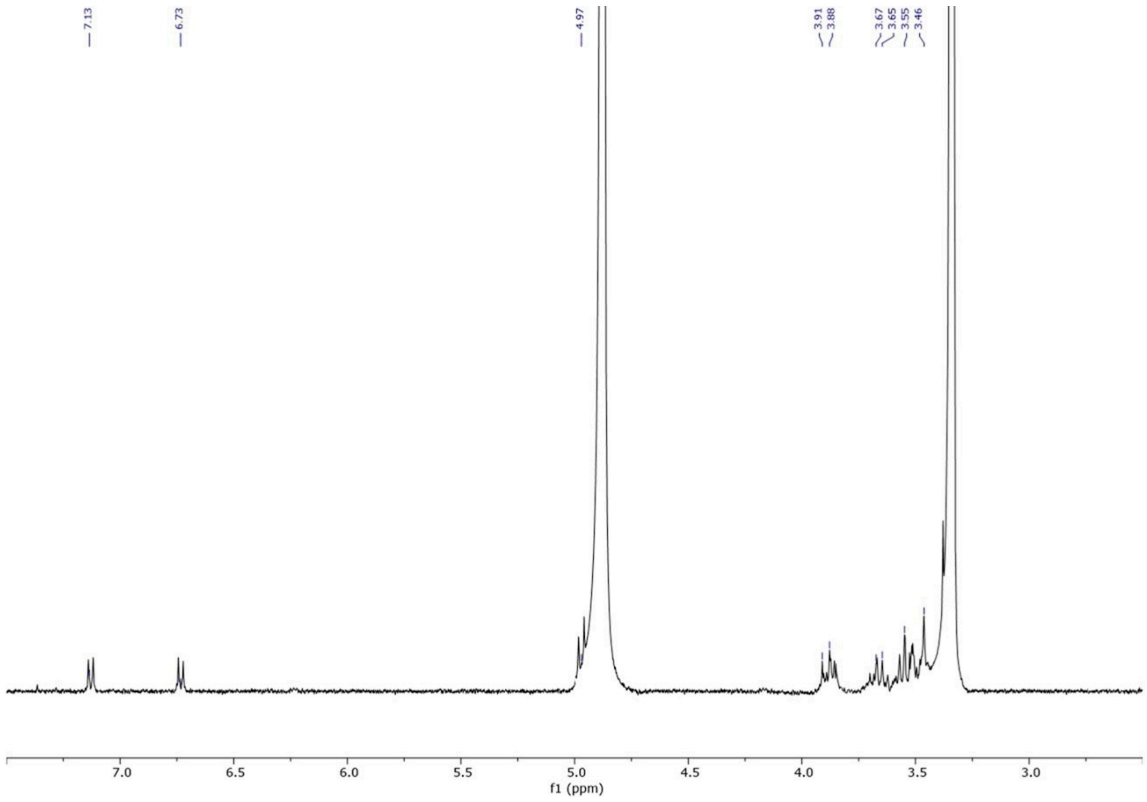


**Figure S10.** $^{1}$H NMR Spectrum (600 MHz, $CD_3OD$) of compound **9.**

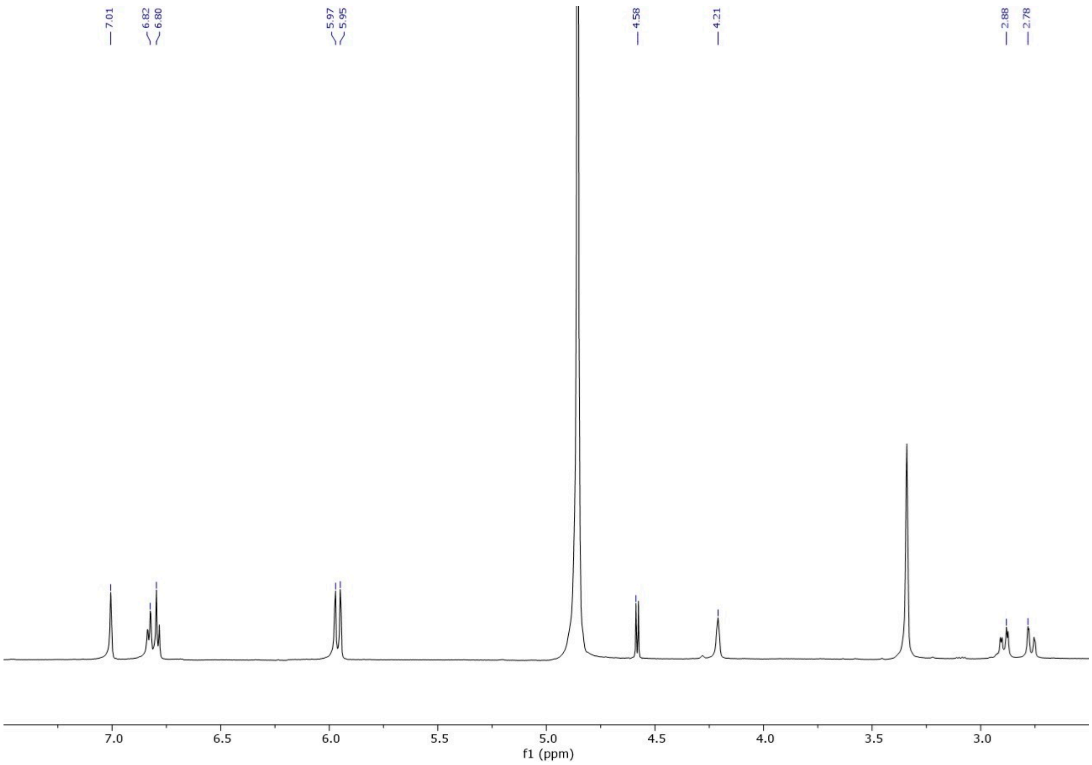


**Figure S11.** $^{1}$H NMR Spectrum (600 MHz, $CD_3OD$) of compound **10.**

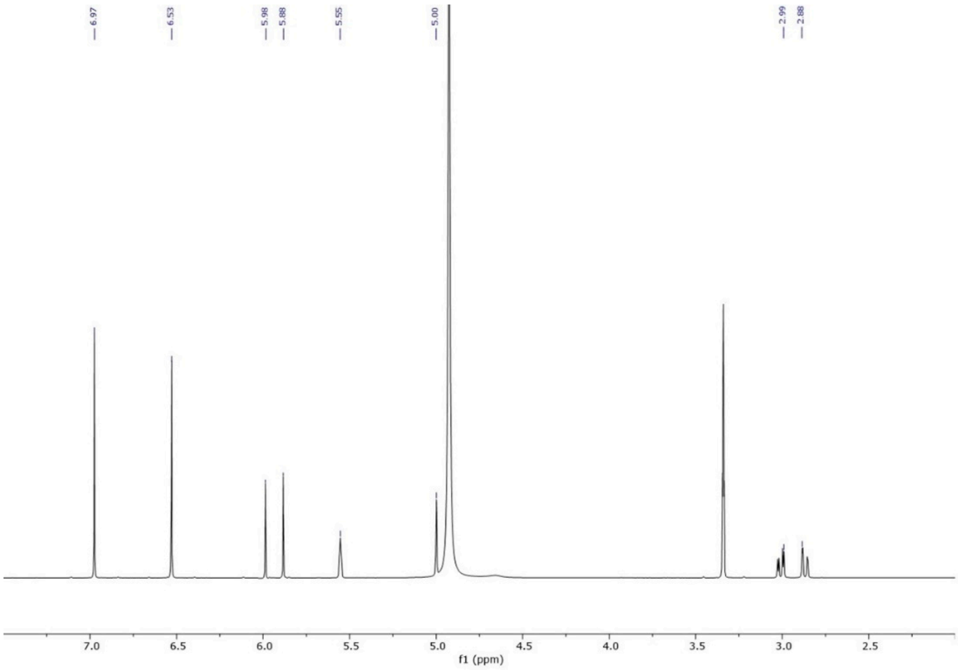


**Figure S12.** $^{1}$H NMR Spectrum (600 MHz, $CD_3OD$) of compound **11.**

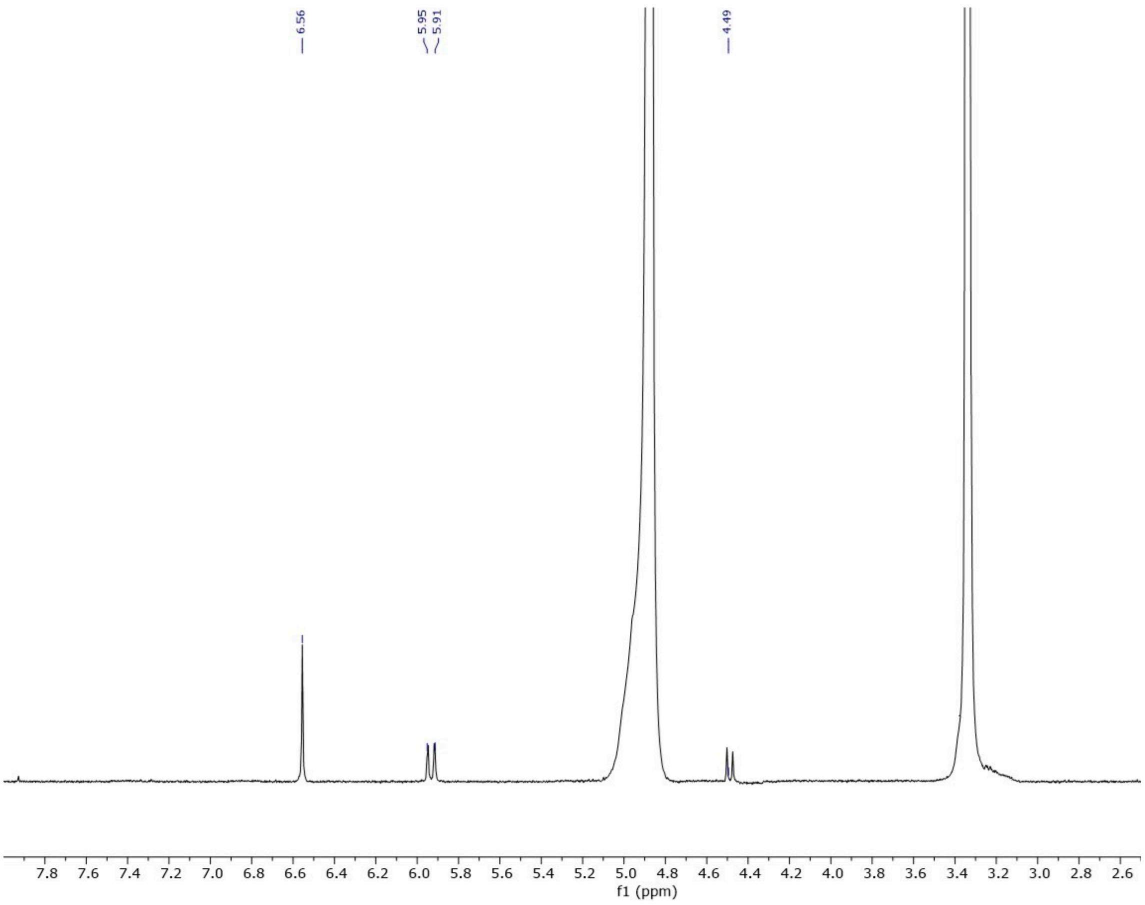


**Figure S13.** $^{1}$H NMR Spectrum (600 MHz, $CD_3OD$) of compound **12.**

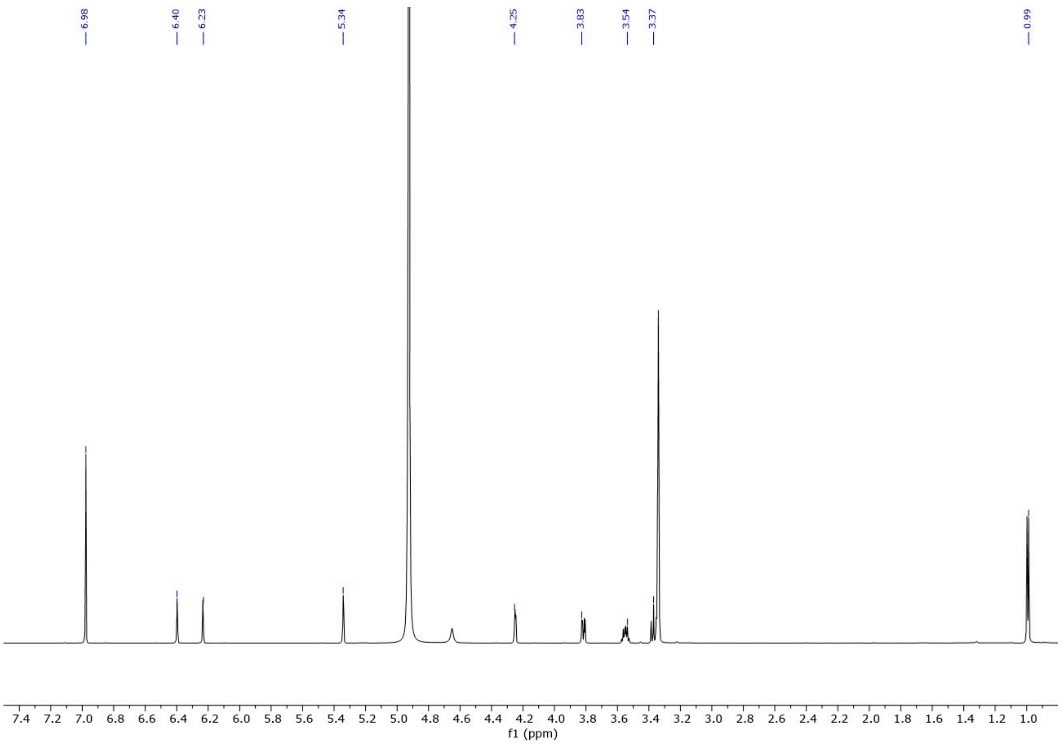


**Figure S14.** $^{1}$H NMR Spectrum (600 MHz, $CD_3OD$) of compound **13.**

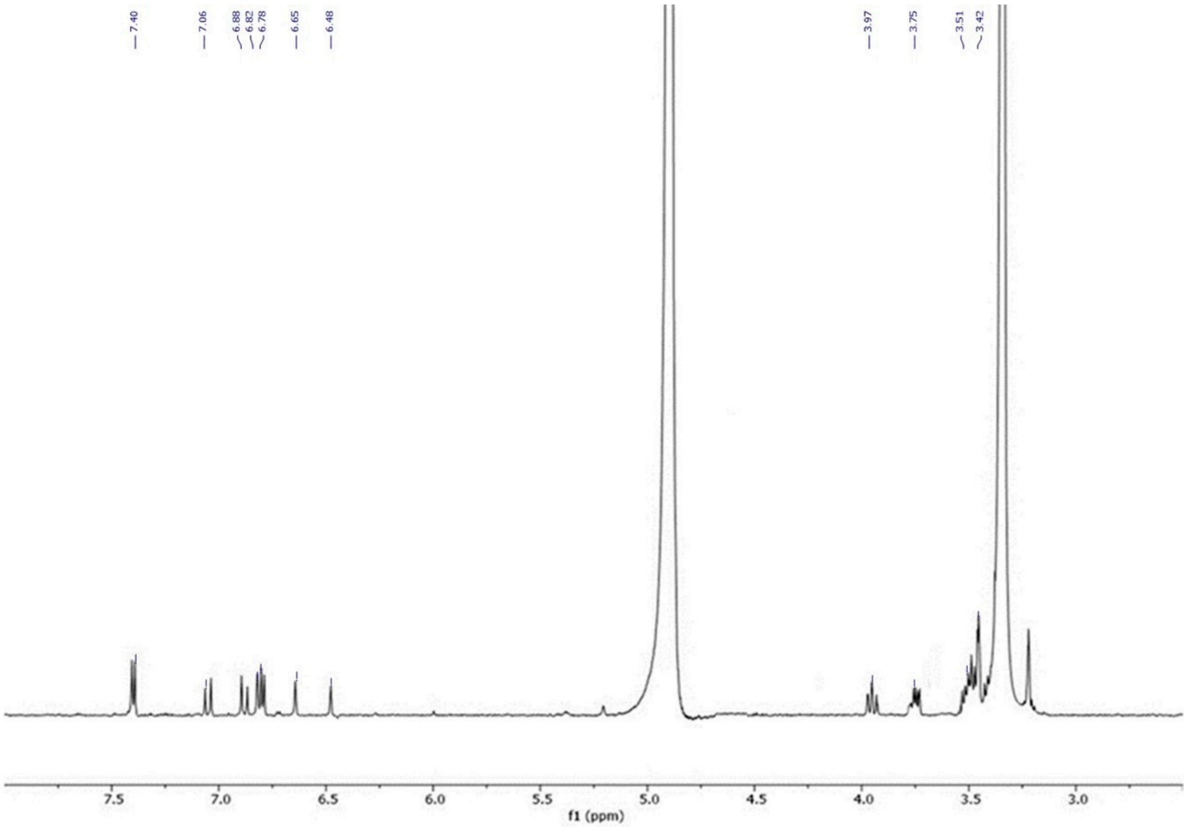


**Figure S15.** $^{1}H$ NMR Spectrum (600 MHz, $CD_3OD$) of compound **14.**

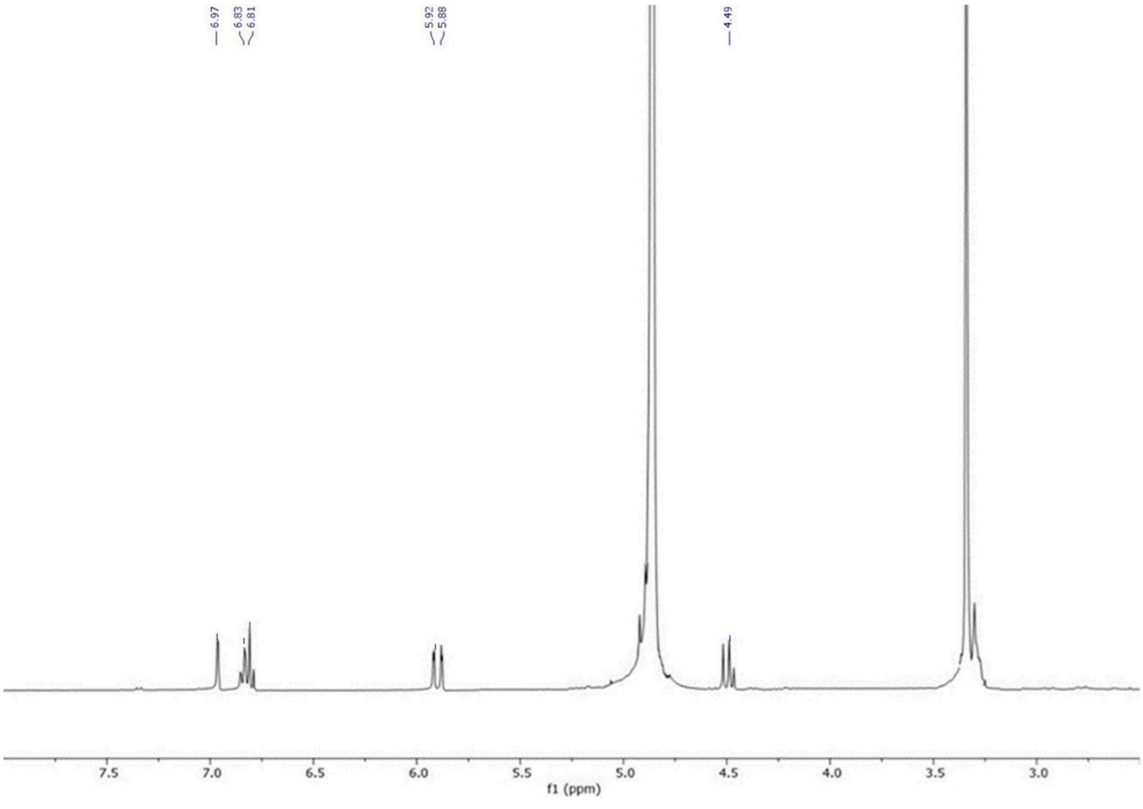


**Figure S16.** $^{1}$H NMR Spectrum (600 MHz, CD3OD) of compound **15.**

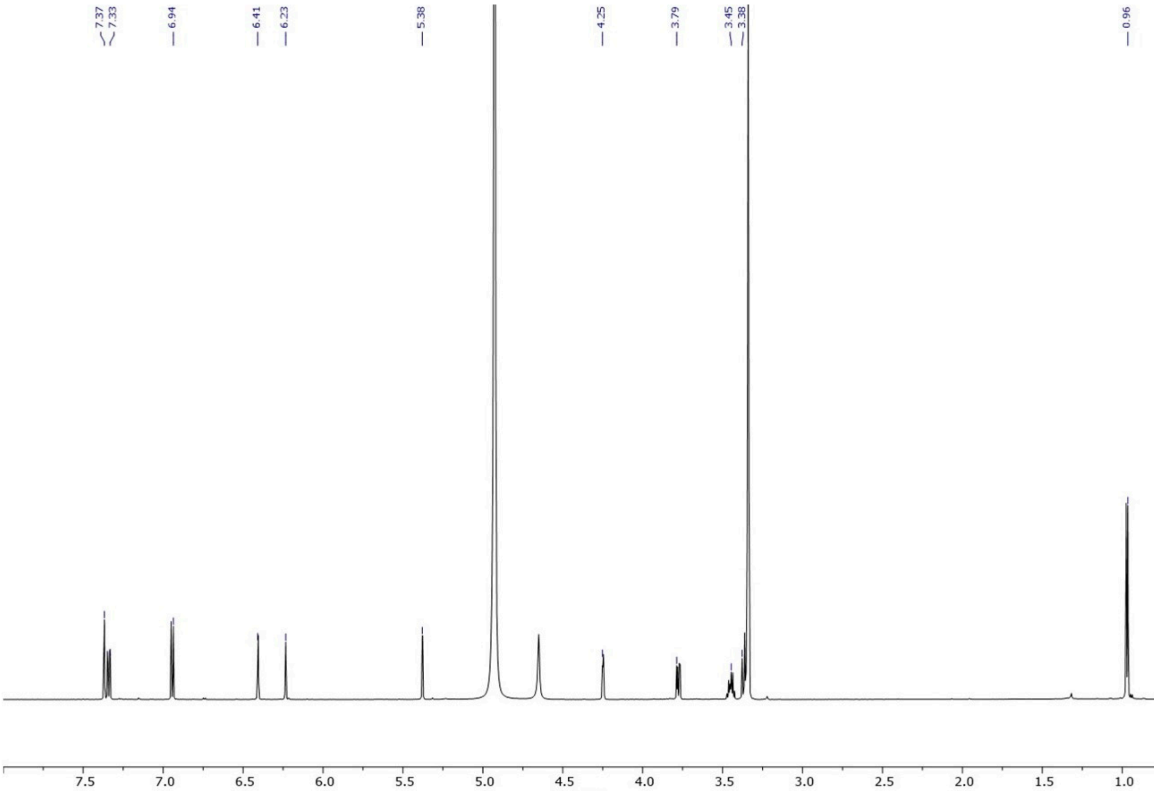


**Figure S17.** $^{1}$H NMR Spectrum (600 MHz, CD3OD) of compound **16.**

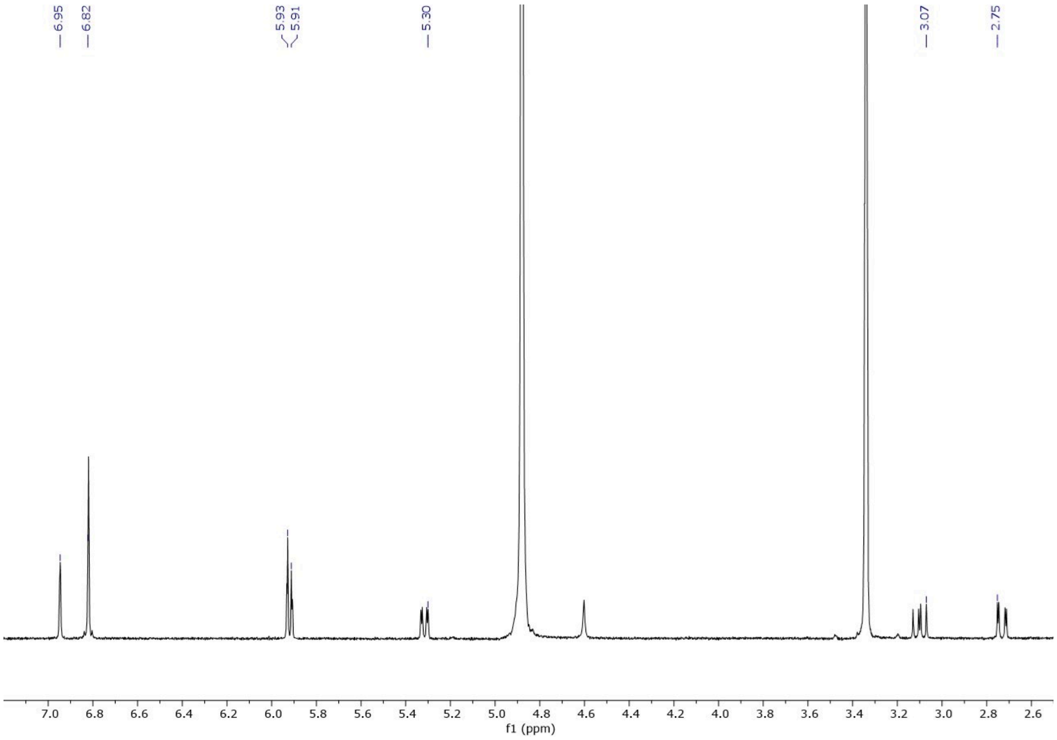


**Figure S18.** $^{1}$H NMR Spectrum (600 MHz, $CD_3OD$) of compound **17.**

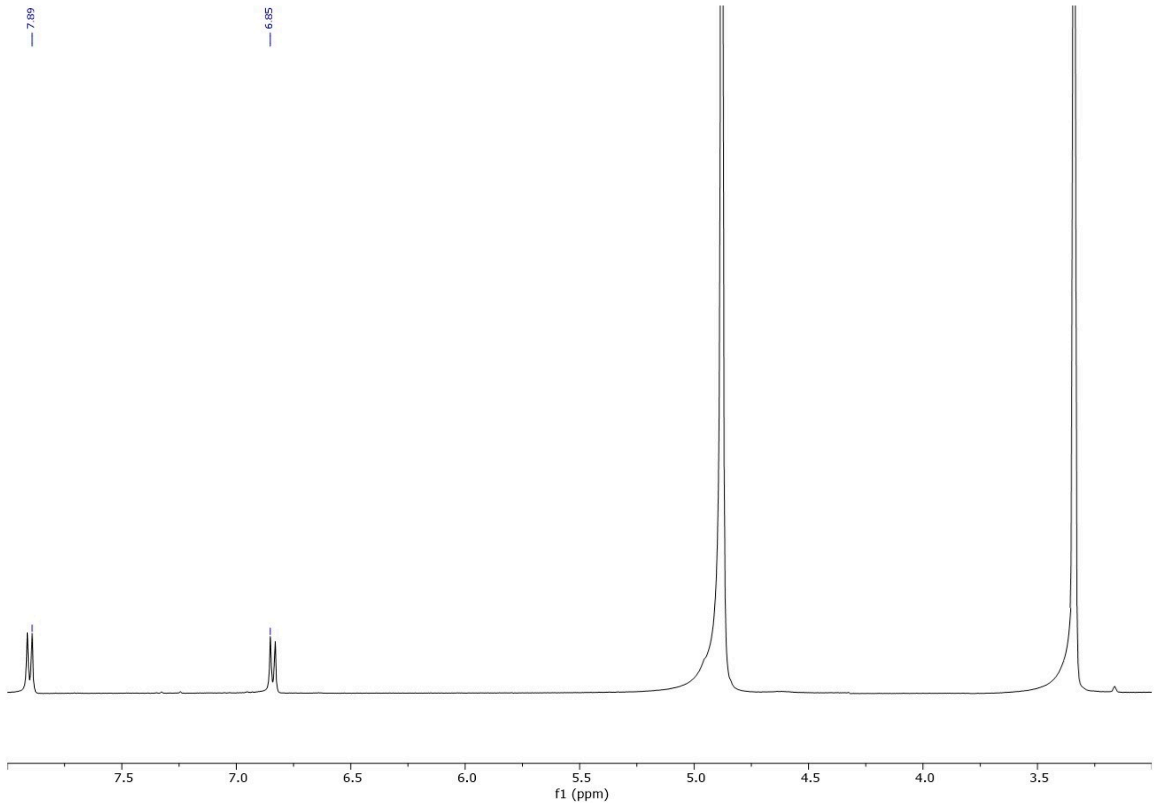


**Figure S19.** $^{1}$H NMR Spectrum (600 MHz, $CD_3OD$) of compound **18.**

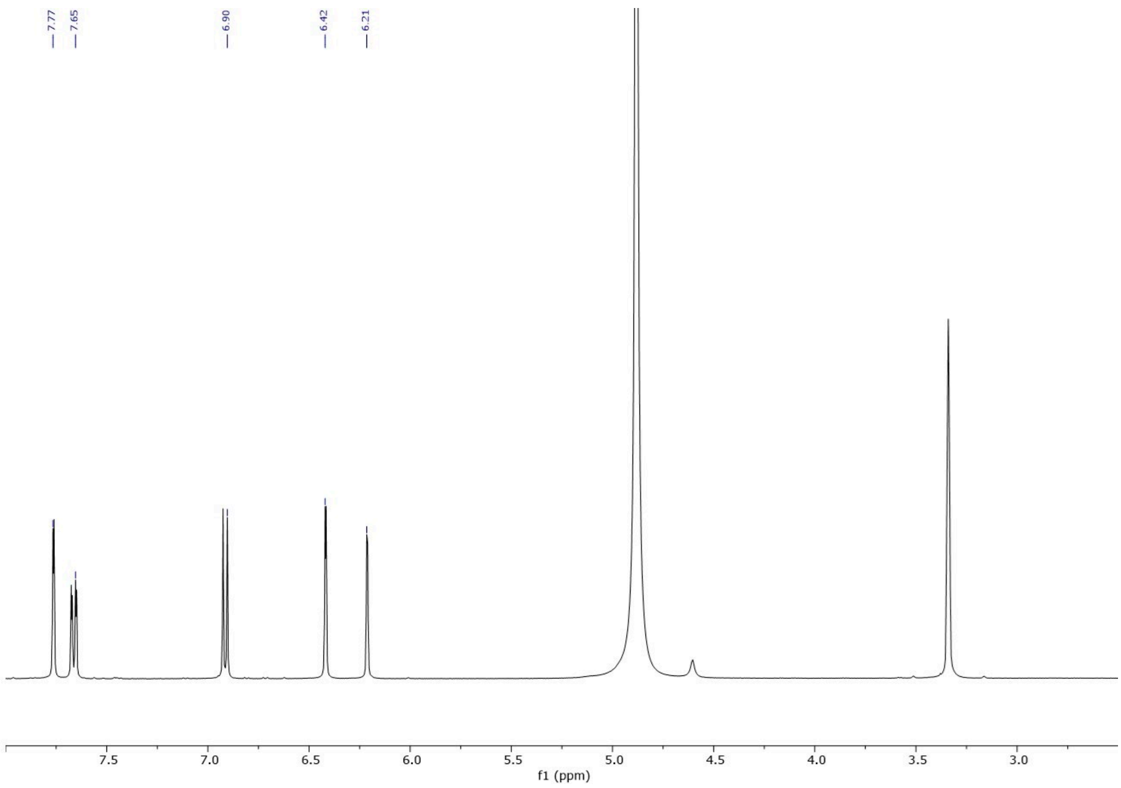


**Figure S20.** $^{1}$H NMR Spectrum (600 MHz, $CD_3OD$) of compound **19.**

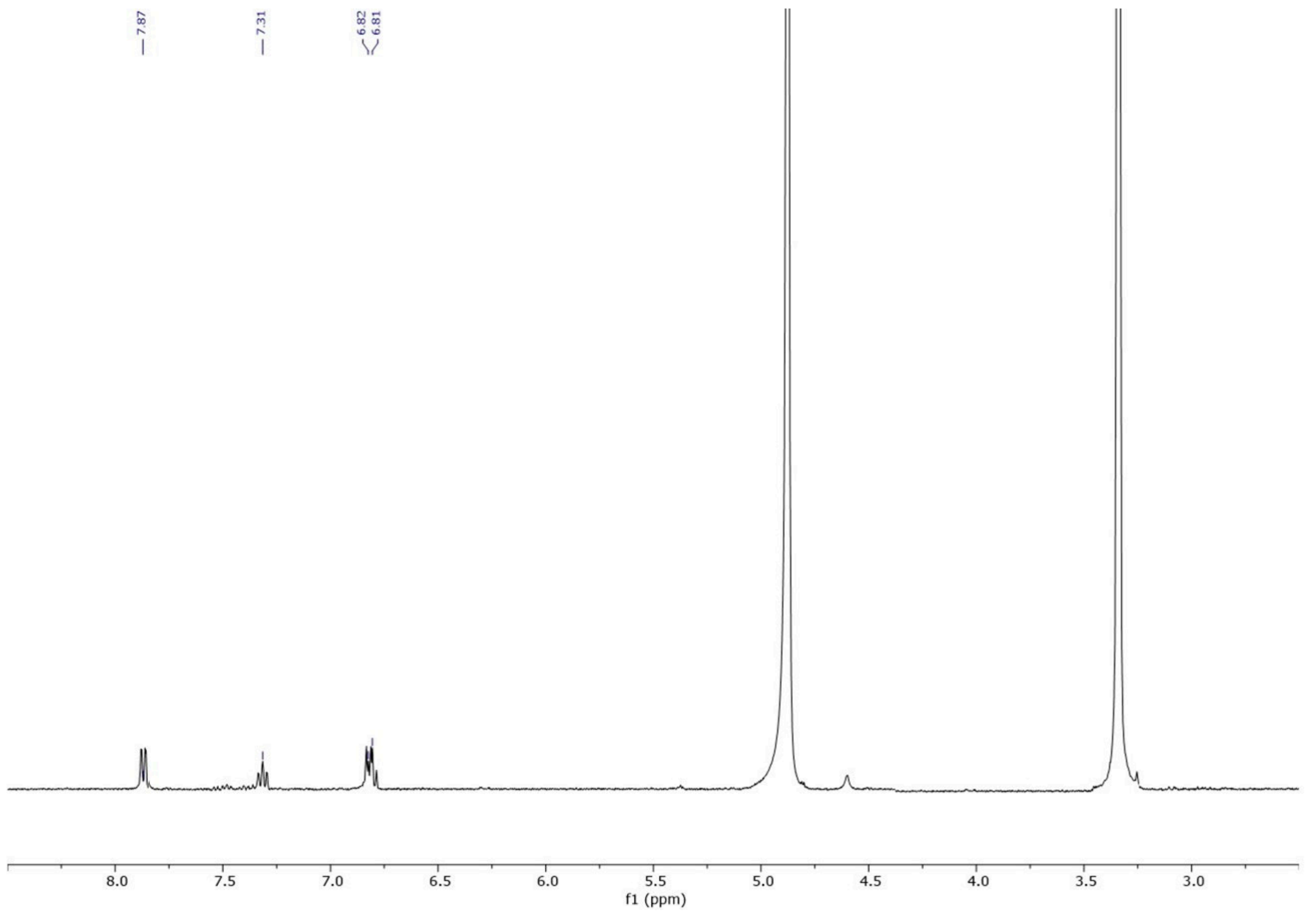


**Figure S21.** $^{1}$H NMR Spectrum (600 MHz, $CD_3OD$) of compound **20.**
